# A dimensional approach to modeling symptoms of neuropsychiatric disorders in the marmoset monkey

**DOI:** 10.1002/dneu.22446

**Published:** 2017-02-20

**Authors:** Lydia Oikonomidis, Andrea M. Santangelo, Yoshiro Shiba, F. Hannah Clarke, Trevor W. Robbins, Angela C. Roberts

**Affiliations:** ^1^ Department of Physiology, Development and NeuroscienceUniversity of CambridgeDowning StreetCambridgeCB2 3DYUnited Kingdom; ^2^ Behavioural and Clinical Neuroscience Institute, University of CambridgeDowning StreetCB2 3EBUnited Kingdom; ^3^ Department of PsychologyUniversity of CambridgeDowning StreetCambridgeCB2 3EBUnited Kingdom

**Keywords:** RDoc, marmoset, neuropsychiatric disorders, prefrontal cortex

## Abstract

Some patients suffering from the same neuropsychiatric disorder may have no overlapping symptoms whilst others may share symptoms common to other distinct disorders. Therefore, the Research Domain Criteria initiative recognises the need for better characterisation of the individual symptoms on which to focus symptom‐based treatment strategies. Many of the disorders involve dysfunction within the prefrontal cortex (PFC) and so the marmoset, due to their highly developed PFC and small size, is an ideal species for studying the neurobiological basis of the behavioural dimensions that underlie these symptoms.Here we focus on a battery of tests that address dysfunction spanning the cognitive (cognitive inflexibility and working memory), negative valence (fear generalisation and negative bias) and positive valence (anhedonia) systems pertinent for understanding disorders such as ADHD, Schizophrenia, Anxiety, Depression and OCD. Parsing the separable prefrontal and striatal circuits and identifying the selective neurochemical modulation (serotonin vs dopamine) that underlie cognitive dysfunction have revealed counterparts in the clinical domain. Aspects of the negative valence system have been explored both at individual‐ (trait anxiety and genetic variation in serotonin transporter) and circuit‐based levels enabling the understanding of generalisation processes, negative biases and differential responsiveness to SSRIs. Within the positive valence system, the combination of cardiovascular and behavioural measures provides a framework for understanding motivational, anticipatory and consummatory aspects of anhedonia and their neurobiological mechanisms. Together, the direct comparison of experimental findings in marmosets with clinical studies is proving an excellent translational model to address the behavioural dimensions and neurobiology of neuropsychiatric symptoms. © 2016 The Authors. Developmental Neurobiology Published by Wiley Periodicals, Inc. Develop Neurobiol 77: 328–353, 2016

## INTRODUCTION

One in five people will suffer from a neuropsychiatric disorder at some point in their life (Kessler et al., 2009), yet the prognosis for successful treatment is still only about 40%. Currently, a handful of pharmacological and psychological therapies are used to treat multiple psychiatric disorders, with the former mostly targeting the widespread monoamine systems in the forebrain (Arroll et al., 2005; Miyamoto et al., 2005). Why is this? First, disorders such as depression and schizophrenia are broadly defined and two individuals may be diagnosed with the same disorder but have no overlapping symptoms, making it unlikely that they will be treated successfully by the same therapeutic strategy. Second, patients diagnosed with different psychiatric disorders may share the same symptom, which could explain why specific pharmacological therapies can be used successfully across multiple disorders. For example, selective serotonin reuptake blockers can be effective in treating patients with general anxiety disorder and depression (Reinhold et al., 2011; Gorman et al., 2014), probably because they target a shared symptom, such as enhanced negative emotion or negative bias. Third, the clinical symptoms are often poorly characterized so two patients may exhibit similar symptoms that actually have different underlying psychological and neurobiological causes. For instance, anxiety may be the result of poor learning of predictive cues signaling negative consequences, causing uncertainty, a known contributor to anxious behavior. Alternatively, impaired attentional flexibility may promote anxiety by making it more likely that subjects stay focused on salient negative stimuli, unable to switch their attention toward more positive events in the environment (Clarke et al., 2015; Shiba et al., 2016). Fourth, even when treatments are successful there is poor understanding of the underlying psychological and neurobiological mechanisms, making it difficult to match specific treatments to specific symptoms in individual patients.

For all these reasons there is growing emphasis in clinical and preclinical studies of therapeutic strategies to target common symptoms regardless of the disorder with which they are associated. Moreover, improved characterization of these symptoms requires a fundamental understanding of the psychological and neurobiological mechanisms that cause them, as recognized by the Research Domain Criteria [RDoc; National Institute of Mental Health, (Insel et al., 2010)]. Although imaging studies of patients suffering from neuropsychiatric disorders have revealed much, both in terms of the neural circuits that appear dysregulated in untreated patients and reversal of this dysregulation following successful treatment, it cannot be ascertained whether this dysregulation is causal or compensatory. Vital for achieving this understanding is animal‐based research, in which experimental manipulations can establish causal relationships and identify the complex interactions between, and within, neural circuits that underlie adaptive and maladaptive behavior. Such research can also identify the neural circuits upon which current therapies act in order to optimize therapeutic targets, eliminate undesirable side effects, and identify new therapeutic targets.

A key brain structure showing altered activity across the range of neuropsychiatric disorders is the prefrontal cortex (PFC) (Strakowski et al., 2005; Shin et al., 2006; Etkin and Wager, 2007; Milad and Rauch, 2007; Koenigs and Grafman, 2009), a multimodal cortical association region with the most extensive reciprocal connections with the rest of the forebrain of all cortical regions (Carmichael and Price, 1996; Ongur and Price, 2000; Petrides and Pandya, 2002; Petrides, 2005; Petrides and Pandya, 2007; Petrides et al., 2012; Yeterian et al., 2012). It is also the only neocortical region that has regulatory control over the brainstem and forebrain chemically specific arousal pathways (Arnsten and Goldman‐Rakic, 1984) which, along with its direct reciprocal connections, gives this region a pervasive influence on perceptual, motoric, attentional, mnemonic, language and emotional systems of the forebrain, both directly and indirectly. Its anatomical and functional organization is relatively preserved across primate species (Ongur and Price, 2000; Burman et al., 2006; Burman and Rosa, 2009; Petrides et al., 2012) making Old World and New World, non‐human primates particularly valuable for translational studies of the prefrontal circuits that underlie the regulation of behavior.

The marmoset, a New World monkey, is ideal for studying the effects of interventions within prefrontal circuits, including their modulation by the monoamine systems. Their brains are relatively small, compared with the much larger brained Old World monkeys, and their cortex, lissencephalic, making it easier to target localized regions of interest cortically and subcortically, either permanently, via fiber‐sparing excitotoxins (e.g., quinolinic acid) and neurochemically specific toxins (e.g., 5,7 dihydroxytryptamine), or temporarily, by infusions of drugs through indwelling cannulae. This will also prove an advantage when applying state‐of‐the‐art molecular and imaging techniques to neural circuit analysis of cognition and emotion, including optogenetics (MacDougall et al., 2016) and pharmacogenetics. Like humans, vision and audition are dominant senses in monkeys, including the marmoset, (in contrast to rodents in which the dominant sense is olfaction), and the expansion of cortical processing of these senses in humans is also seen in non‐human primates (Orban et al., 2004; Rauschecker and Scott, 2009). In addition, compared with rodents, marmosets show distinct gene expression patterns in the visual and prefrontal cortex despite similarities in genetic markers in many other areas (Mashiko et al., 2012). This makes primates ideal for translational studies of higher‐order cognitive and affective processes using behavioral tests that rely on these dominant senses. An additional advantage of marmosets is that it is possible to maintain purpose bred colonies within spacious accommodation at local institutions as a consequence of their small size and their ease of breeding in captivity. This allows for the necessary large scale studies of neural circuits. Maintaining a large breeding colony allows the investigation of the interaction between genetic and behavioral traits that are known risk factors for neuropsychiatric disorders, for instance, the serotonin transporter polymorphism and high trait anxiety. The onset of many neuropsychiatric disorders occurs during childhood and adolescence (Jones, 2013) and 75% of adults suffering from a mental disorder have an onset before the age of 25 (Kessler et al., 2005). Thus, the short 5 month gestation period of marmosets and the fact that they reach adulthood by 2 years (Abbott and Hearn, 1978; Abbott et al., 2003; Schultz‐Darken et al., 2016) makes them the ideal primate species in which to study the normal and abnormal development of prefrontal circuits related to these genetic and behavioral risk factors.

**Figure 1:**
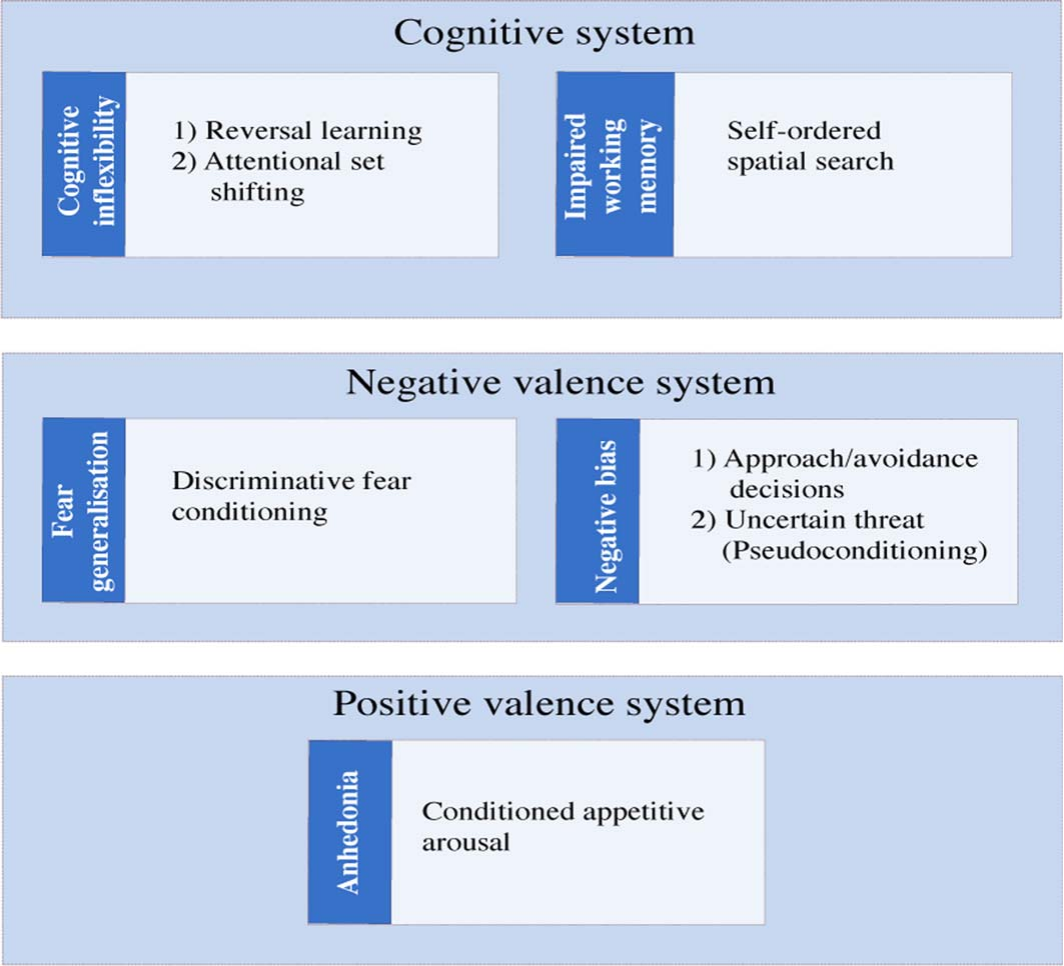
RDoc in the marmoset. Tasks developed in the marmoset are represented in white, linked to the relevant behavioral dimension that is impaired across disorders (blue) and placed within the appropriate system (Cognitive, Negative Valence, and Positive Valence, light blue). [Color figure can be viewed at wileyonlinelibrary.com]

In this review we will focus on a number of behavioral dimensions common to a variety of neuropsychiatric disorders and describe the range of cognitive and affective tests that have been developed to study their psychological and neurobiological bases in the marmoset. We will include dimensions associated with dysfunction in the cognitive (cognitive inflexibility and working memory), negative valence (fear generalization and negative bias) and positive valence (anhedonia) systems, as defined by RDoC (Fig. 1). Since symptoms are core to neuropsychiatric diagnoses and are often the trigger for individuals to seek help and advice from a clinic, knowledge of the relationship between behavioral dimensions and symptomatology is critical for progress in our understanding of the etiology and treatment of these disorders.

## COGNITIVE SYSTEM DYSFUNCTION, COGNITIVE INFLEXIBILITY, AND IMPAIRED WORKING MEMORY

Impairments within the cognitive systems are prominent in disorders such as schizophrenia, obsessive‐compulsive disorder (OCD) and attention deficit hyperactivity disorder (ADHD). These disorders may share a range of cognitive impairments including deficits in cognitive flexibility and inhibitory response control and aspects of working memory (Morice, 1990; Chamberlain et al., 2005; Castellanos et al., 2006). Such deficits may also be present in affective disorders, including anxiety and mood disorders (Airaksinen et al., 2005; Mantella et al., 2007; Rock et al., 2014). Numerous behavioral tasks have been developed to study cognitive flexibility and working memory abilities in marmosets. Here we focus on those tests that have been successfully translated into clinical and pre‐clinical studies in humans, and back translated into rodents. Specifically, cognitive flexibility has been measured using discrimination reversal‐learning and attentional set shifting tasks, whilst working memory and its underlying mechanisms have been studied using a spatial self‐ordered sequencing task (Fig. 1). The translational success of these tasks for modeling the overlapping deficits in behavioral dimensions and symptoms across disorders has already provided enormous insight into the separable prefrontal circuits and neurochemically specific modulation that underlie such dimensions.

Cognitive flexibility is the ability to adapt mental strategies and actions to the changing contingencies and conditions of the environment (Cañas et al., 2003). It has been suggested that cognitive inflexibility underlies the compulsions present in OCD patients as they shift from flexible, goal directed actions to persistent maladaptive habitual behaviors (Graybiel and Rauch, 2000; Gillan et al., 2011). Although cognitive inflexibility has also been linked to perseverative thinking and the inability to perform mental shifts in schizophrenia (Delahunty et al., 1993; Elliott et al., 1995; Pantelis et al., 1999), it is unclear whether it underpins these symptoms or is simply a by‐product of the prefrontal dysfunction associated with the disorder (Orfei et al., 2013; Weinberger and Berman, 1996). Using the Wisconsin Card Sorting task (WCST, Berg, 1948; Milner, 1963) a commonly used clinical test to evaluate set‐shifting ability in patients with frontal lobe damage, some studies have shown that OCD patients display marked impairments in shifting attentional sets (Okasha et al., 2000; Fontenelle et al., 2001; Veale et al., 2009); but see (Abbruzzese et al., 1995, 1997; Purcell et al., 1998; Simpson et al., 2006). In one such study the level of the impairment correlated with the severity of the symmetry/ordering obsessions (Lawrence et al., 2006) suggesting that difficulties in shifting attention may contribute to the development of these obsessions. Similar difficulties have also been reported not only in patients suffering from schizophrenia (Canavan et al., 1989; Beatty and Monson, 1990) but also Huntington's disease (Malmo, 1974; Lysaker et al., 1995; Everett et al., 2001).

**Figure 2:**
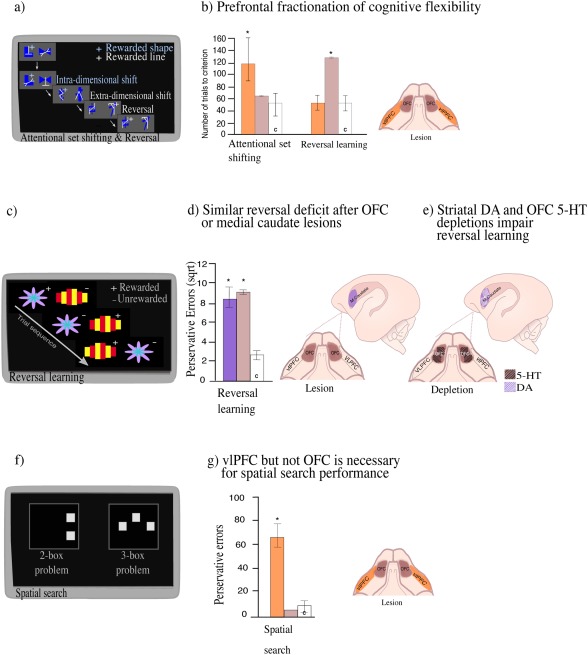
Studying cognitive system dysfunction in the marmoset. (a) Schematic of the attentional set shifting and reversal learning paradigm. In Intra‐dimensional shifts, new exemplars, still varying along the same two perceptual dimensions of shapes and lines are presented and the previously relevant dimension for example, shape, remains relevant and animals must learn which of the two new exemplars from that dimension is rewarded. In extra‐dimensional shifts the relevant dimension is changed (e.g., shape to line) and an exemplar from the new relevant dimension is now rewarded. In contrast, in reversal, the exemplars and dimensions remain the same but the rewarding contingency is reversed (e.g., shape A to shape B). (b) Number of trials required to reach criterion performance during an extra‐dimensional shift (attentional set shifting) and reversal after antOFC or vlPFC excitotoxic lesions. Bars represent the range of scores in each group (Dias et al., 1996a, b). (c) Schematic of the serial reversal learning paradigm, showing an example set of three trials. (d) Perseverative errors (consecutive responses to the unrewarded exemplar, square‐root transformed) made across four reversals after excitotoxic lesions of antOFC or the medial caudate nucleus (Clarke et al., 2008). Error bars represent SEM. (e) Depletions of 5‐HT in the OFC and DA in the medial caudate selectively impair reversal learning. (f) Schematic of the self‐ordered spatial search task with 2‐box and 3‐box examples. The locations of the boxes change across trials. (g) Number of perseverative errors (consecutive responses to the same spatial location) made after antOFC or vlPFC excitotoxic lesions (Walker et al., 2009). Error bars represent SEM. In all figures the location of the excitotoxic lesions are shown on the diagram of the orbital (antOFC, vlPFC) or sagittal (medial caudate) view of the brain. White bars (C) represent control group and stars (*) represent *p* < 0.05.

In order to dissect out the underlying cognitive deficits that may contribute to impaired WCST performance, which may include disrupted motivation, attention, learning and memory, a multidimensional discrimination task was developed for use in both humans and marmosets (Roberts et al., 1989). The task was based on intra‐dimensional (ID) and extra‐dimensional (ED) shift studies of animal learning (Mackintosh and Little, 1969). It required subjects to attend to the different aspects of multidimensional stimuli (varying in shape and lines for instance) and either attend to the same dimension across discriminations (intra‐dimensional shift) or shift attention from one dimension to another [extra‐dimensional shifts; Dias et al., 1996a, b; Fig. 2a]; the latter is a direct parallel of the switch from sorting cards according to one category, to sorting them according to another, that is at the core of the WCST. The test also enabled direct comparison with another type of cognitive flexibility that had commonly been studied in primate neuropsychological studies (Jones and Mishkin, 1972), namely reversal learning. Here, having learned to respond to one of two stimuli in order to receive reward, subjects had to reverse their responding to the other, previously unrewarded stimulus in order to gain reward.

By directly comparing the effects of excitotoxic lesions of two distinct PFC regions, namely the orbitofrontal cortex (OFC) and ventrolateral prefrontal cortex (vlPFC), studies in marmosets revealed their differential contribution to these two forms of cognitive flexibility (Dias et al., 1996a, 1996b). While OFC lesions disrupted reversal learning but not attentional set‐shifting, vlPFC lesions disrupted attentional set‐shifting but not reversal learning [Fig. 2(b)], implicating the former in monitoring the changing affective value of stimuli in the environment and the latter in attentional control. This double dissociation was subsequently replicated in human functional neuroimaging studies (Hampshire and Owen, 2006) as well as in rats (Birrell and Brown, 2000) and mice (Bissonette et al., 2008), highlighting the forward and back translatability of these findings. Further studies in the marmoset have implicated the medial caudate nucleus in reversal learning (Clarke et al., 2008) with the pattern of impairment similar to that seen following OFC lesions. In both cases, animals displayed perseverative responding, whereby the previously rewarded option is chosen repeatedly, despite the lack of reward.

Marked differences in the underlying monoaminergic modulation of these circuits have also been revealed. Reductions in dopamine, but not serotonin affect higher‐order attentional selection in the vlPFC (Roberts et al., 1994; Clarke et al., 2005). In contrast, reductions of serotonin, but not dopamine, within the OFC mimic the perseverative effects of excitotoxic OFC lesions on reversal learning (Clarke et al., 2004, 2005, 2007). The converse, however, is the case at the level of the caudate nucleus where reversal learning is impaired following reductions in dopamine but not serotonin (Clarke et al., 2011). Thus, distinct neurochemical systems at the level of the striatum and OFC regulate reversal learning, and although both serotonin and dopamine pathways richly innervate the PFC, their contributions differ with respect to the different forms of cognitive flexibility [Clarke et al., 2008; Fig. 2(e)].

Together these findings provide considerable insight into the specific fronto‐striatal circuitry subserving cognitive flexibility and are beginning to reveal how these different forms of cognitive flexibility contribute to disorders such as schizophrenia and OCD. For example, ED shifting, as assessed in the CANTAB (Cambridge Neuropsychological Test Automated Battery), generally shows a significant impairment in OCD patients (Watkins et al., 2005; Chamberlain et al., 2006). This deficit appears to be pre‐symptomatic as it is also present in their healthy, first degree relatives (Chamberlain et al., 2007). This may implicate altered functioning in vlPFC in OCD patients and a recent report specifically links attentional set‐shifting performance and vlPFC function. Specifically, decreased functional connectivity between vlPFC and caudate at resting state was linked to impairments in attentional set‐shifting performance and predicted greater number of errors during ED shifts (Vaghi et al., 2016). In contrast, OCD patients display mild or no detectable impairment in reversal learning. However, they do show a speed‐for‐accuracy trade off that has been correlated to the severity of compulsions (Chamberlain et al., 2007, 2008; Valerius et al., 2008). Moreover, at the level of underlying brain circuitry, OCD patients and their unaffected first‐degree relatives display decreased recruitment of the OFC during reversal learning (Chamberlain et al., 2007; Remijnse et al., 2013) and symptom provocation (Morgiève et al., 2014) and abnormalities in resting state activity in this region (Menzies et al., 2008). A meta‐analysis of imaging studies highlights not only the OFC but also vlPFC regions as areas with increased likelihood of activation in response to symptom provocation (Rotge et al., 2008). Thus, altered activity in both OFC and vlPFC circuitry involved in response reversal and rule shifting, respectively, may underlie the impairments in cognitive flexibility in OCD and contribute to the compulsions and obsessions, respectively.

Similarly, patients with schizophrenia also show marked deficits in shifting attentional sets (Elliott et al., 1995; Morris et al., 1999; Pantelis et al., 1999; McKirdy et al., 2009) and in reversal learning (Thoma et al., 2007; Waltz and Gold, 2007; Leeson et al., 2009). Performance on the latter is related to the severity of the negative symptoms (Pantelis et al., 1999; Leeson et al., 2009), which if they persisted were linked to greater impairment in set shifting at follow‐up (Leeson et al., 2009). This pattern of impairments also implicates dysfunctional OFC and vlPFC circuits in the cognitive inflexibility associated with schizophrenia, similar to that proposed for OCD. However, the specific neural basis of these deficits across the disorders remains to be determined, given that, for example, similar perseverative responding in reversal learning is associated with disrupted OFC or medial striatal activity (Dias et al., 1996a; Clarke et al., 2008).

In schizophrenia, support for dysfunction within the medial striatum is provided by evidence for disturbances in activation and connectivity within this region (Vink et al., 2006; Rolland et al., 2015) and the finding that reduced functional activity within the ventral striatum is associated specifically with impaired reversal performance in unmedicated patients compared with controls (Schlagenhauf et al., 2014). This can be contrasted with the decreased recruitment of the OFC during reversal learning in OCD patients described above. It can be argued that given vlPFC and OFC form part of a neural network to control attentional set‐shifting and reversal learning respectively, dysregulation at any node within the network will disrupt its overall functioning. However, identifying the underlying cause of that disruption has important implications for the development of therapeutic strategies for the two disorders. For example, studies in the marmoset have revealed that the OFC and striatal mechanisms that underlie reversal learning in the marmoset are differentially modulated by serotonin and dopamine, respectively. Thus, the improvement in OFC function and symptoms in OCD patients following administration of selective serotonin reuptake inhibitors (SSRIs) (Nakao et al., 2005; Saxena et al., 2009) would be predicted by the selective actions of serotonin on reversal learning within the marmoset OFC. It may also explain why medicated OCD patients do not show errors in reversal performance as the boosting of serotonin with SSRI treatment may alleviate their reversal performance deficits. In contrast, pharmacotherapies targeting the dopamine system (rather than SSRIs) would be predicted to alleviate the striatal‐related reversal deficits in schizophrenia. Although both typical (Michara and Goldberg, 2004) and atypical (Keefe et al., 1999) antipsychotics primarily targeting dopaminergic function have been linked to general improvements in cognitive function, whether schizophrenia patients specifically benefit from antipsychotic medication in terms of improvements in striatal based cognitive flexibility remains to be determined.

Within the domain of cognitive symptoms, schizophrenia patients also display impairments in working memory (Green, 1996), which are also a core symptom of attention deficit/hyperactivity disorder (ADHD) (Westerberg et al., 2004; Lee and Park, 2005; Martinussen et al., 2005). Working memory is the ability to hold information “on‐line” in memory, to update that information across time and to use that information to guide responding. A variety of tests has been used to study working memory including delayed response tasks (Pontecorvo et al., 1996), multi‐arm mazes (commonly used in rodents; Dudchenko et al., 2013) and self‐ordered search tasks (Petrides, 1995a, 1995b). An example of the latter developed in the marmoset is conceptually very similar to the CANTAB self‐ordered spatial search task used to study working memory in patients (Owen et al., 1990; Manes et al., 2002; Luciana, 2003; Chase et al., 2008) It requires a combination of working memory, response inhibition and strategy implementation abilities [Fig. 2(f)]. Marmosets are presented with squares simultaneously placed in two, three, or four spatial locations (out of eight possible combinations) on a touch‐sensitive computer screen. On all trials, marmosets are required to respond once only to each square in a self‐ordered sequence in order to receive reward (Collins et al., 1998; Walker et al., 2009). In the marmoset, excitotoxic lesions of the vlPFC, but not OFC, dramatically impair performance [Walker et al., 2009; Fig. 2(g)]. Marmosets are unable to attain pre‐lesion levels of performance and exhibit high numbers of perseverative responses, as they touch the same square repeatedly, failing to move on to the next, previously untouched square. However, performance can be rescued by removing the previously touched square from the touchscreen until another response has been made, thereby preventing perseveration of the immediately preceding response. This suggests that the inability to solve this task stems from a tendency to repeat previous responses.

In the human version of the task, patients with schizophrenia and ADHD patients exhibit similar impairments. Both adults (Dowson et al., 2004) and children (Fried et al., 2015) with ADHD exhibit a profound impairment in this task. Moreover, schizophrenia patients in particular fail to adopt a systematic strategy and make numerous between‐search errors, performing worse than frontal lobe patients (Pantelis et al., 1999; Badcock et al., 2005) and being impaired even at first‐episode psychosis (Joyce, 2002; Joyce et al., 2005). This was evident even when controlling for low visuo‐spatial memory span, suggesting that impaired executive function and strategy implementation are at the core of the working memory problem. Deficits in spatial working memory extend to the healthy monozygotic twins of patients (Pirkola et al., 2005) or first degree relatives (Wood et al., 2003). This would suggest that the working memory impairment is an endophenotype of schizophrenia.

Whilst evidence from the clinical literature supports global prefrontal impairment in ADHD (Lenartowicz et al., 2014; Arai et al., 2015; Mattfeld et al., 2015) and schizophrenia patients (Manoach, 2003; Orfei et al., 2013; Pu et al., 2013; Marumo et al., 2014; Buchy et al., 2015), the identification of the vlPFC as critical for self‐ordered spatial memory in marmosets specifically implicates vlPFC impairment in this working memory deficit in patients. This is supported by the accompanying profound dysfunction in attentional set shifting, which also is associated with vlPFC dysfunction. Similarly, children (Kempton et al., 1999) and adult (Clark et al., 2007) ADHD patients that exhibit marked impairment in the self‐ordered spatial search task also show impaired response inhibition. Such deficits in working memory can be ameliorated by either domain specific cognitive training or psychostimulant medication (Kempton et al., 1999; Bedard et al., 2004; Mehta et al., 2004; Klingberg et al., 2002, 2005; see Del Campo et al., 2011), the latter implicating the dopamine or noradrenaline systems. In support of the dopamine system, administration of a D1 agonist improves a ketamine‐induced deficit on the spatial‐order search task in marmosets (Nakako et al., 2013) suggesting that the working memory processes in this task are under dopaminergic modulation. However, given that striatal dysfunction is also implicated in these disorders (Durston et al., 2003; Cubillo et al., 2012 for a review) it remains to be determined whether the improvements are due to the actions of drugs at the level of the PFC or striatum (see, e.g., Clatworthy et al., 2009).

In summary, the marmoset is proving an excellent model in which to parse the neural circuitry and neurochemical pathways that underlie the cognitive symptomatology associated with a range of neuropsychiatric disorders including OCD, schizophrenia, and ADHD. This is an important first step toward stratification of specific symptoms both within, as well as between, disorders. Moreover, identification of the level within a particular circuit from which a cognitive impairment may arise, for example, PFC or striatum, and the specificity of the neurochemical modulation of that level within the circuit, for example, dopamine or serotonin, has important implications for targeting current therapies more effectively. Future studies employing viral mediated vectors to target specific pathways using optogenetics or pharmacogenetics can further delineate the circuit and the marmoset is an ideal model for such studies with a highly differentiated prefrontal cortex but a relatively small and lissencephalic brain.

## DYSREGULATION OF NEGATIVE VALENCE SYSTEMS, ATTENTIONAL BIASES, AND FEAR GENERALIZATION

Although there are evolutionary advantages to fear and anxiety (they can act as a protective defense mechanism in dangerous situations), they can easily become maladaptive and deleterious once left unregulated. Pathological anxiety, affecting more than 28% of the general population (Kessler et al., 2009), specific phobias, PTSD, abnormally low mood in depression and disturbances of emotion in schizophrenia (Staring et al., 2009) are all a result of a dysregulated negative valence system. In generalized anxiety disorder, patients exhibit pathological worry and apprehension over long periods of time about situations that are normally causing no distress to the healthy population (Tyrer and Baldwin, 2006; Craske et al., 2009). In specific phobias and PTSD the worry and distress are focused on, and triggered by, a single event, situation or stimulus (Kessler et al., 2009; Pacella et al., 2013).

A particularly prominent symptom in patients suffering from all types of anxiety and depression is fear over‐generalization, whereby they indiscriminately develop fearful responses to threatening and non‐threatening stimuli alike (Lissek et al., 2008; Craske et al., 2009; Lissek et al., 2014). They also tend to develop negative biases, not only within the affective domain but also extending into the cognitive domain, and influencing attention and memory (Murphy et al., 1999; Gotlib et al., 2004; Mogg and Bradley, 2005; Bar‐Haim et al., 2007). It is still unclear whether fear generalization and negative biases are causal to the development of the disorder, or constitute underlying symptoms that appear in its presence and contribute to its maintenance. Evidence for the former comes from the study of individuals within the healthy population who display quite dramatic differences in anxiety. This variation in anxiety is recognized as a stable personality trait. Individuals at the extreme high end of this trait can be more at risk of developing pathological anxiety and, like individuals suffering from clinical anxiety, have a tendency to over‐generalize in fear provoking situations and display mild negative biases (Sexton et al., 2010; Arnaudova et al., 2013). Such behavioral traits are the product of interactions between genes and early life experiences (Nugent et al., 2011). Life experiences that may trigger the development of these traits include physical and psychological stressors, which impact on the development of brain circuits underlying emotion. Indeed, affective disorders commonly emerge during childhood and adolescence (Jones, 2013), making the study of gene–environmental interactions and brain development important for our understanding of emotion dysregulation.

The common marmoset, like humans, displays marked individual responsivity to anxiety provoking situations that appears stable across time and thus trait‐like in character. This makes the marmoset an ideal species for studying emotion regulation and dysregulation. Consequently, a range of specific tasks have been developed to formally characterize their anxious temperament and to measure fear over‐generalization and negative biases (Fig. 1). An important aim in developing these tasks has been to bridge the gap between existing studies of negative emotion in rodents, monkeys and humans. The neural mechanisms underlying fear and anxiety have been extensively studied in rodents (LeDoux, 2000; Maren, 2001; Davis et al., 2010; Tovote et al., 2015), primarily using Pavlovian fear conditioning paradigms (acute and sustained fear) and tests of innate anxiety, such as the open field and elevated plus maze (Belzung and Griebel, 2001). In contrast, specific tests of innate fear (responsivity to snakes) and anxiety (responsivity to unknown humans) have been more commonly used in non‐human primates (Izquierdo et al., 2005; Rudebeck et al., 2006; Kalin et al., 2007; Machado and Bachevalier, 2008) making cross‐species comparison and translation difficult. Moreover, although fear conditioning has been used to test both humans and rodents alike, the metric of emotion differs between the two species; a behavioral freezing response is commonly measured in rodents contrasted to an autonomic and/or self‐reported state used in humans. Since an emotional state is the product of changes across the range of outputs, both behavioral and physiological, it is important to measure multiple aspects of the response in order to determine the neural circuits that regulate emotional states. This is particularly important since there are strong brain‐body‐brain pathways resulting in patients with anxiety and mood disorders being more likely to suffer cardiovascular disease and vice versa (Khawaja et al., 2009; Stapelberg et al., 2012).

**Figure 3:**
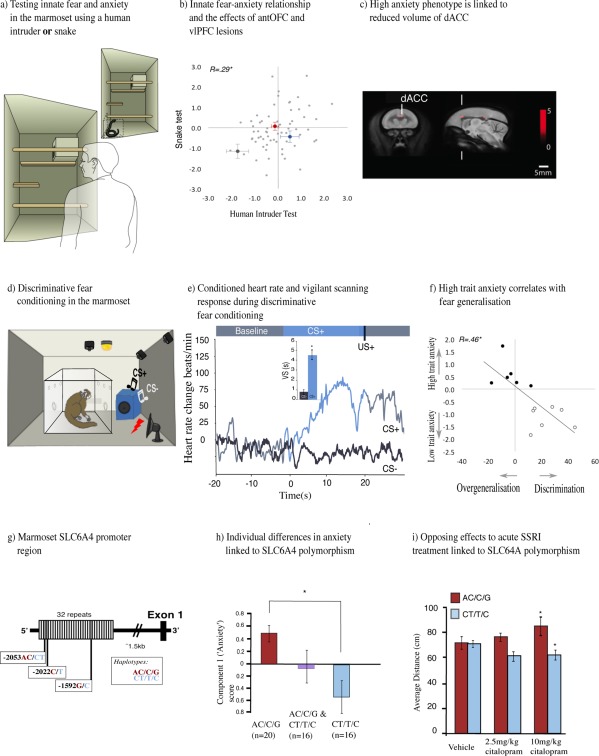
Dysregulation of negative valence systems. (a) Human intruder and snake tests are conducted in the home‐cage, with the animal separated from their cage mate in one quadrant of the cage for a brief period. (b) The negative emotional response to the snake, as measured by the component 1 (“Anxiety”) score in the Principal Component Analysis (PCA), is positively correlated with the negative emotional response to the human intruder. AntOFC (red) and vlPFC (blue) excitotoxic lesions increase negative emotion relative to controls (gray) but the lesioned animals remain within the normal variation of trait anxiety within the colony (Agustín‐Pavón et al., 2012). (c) Two clusters (dACC, labeled, and posterior cingulate cortex) identified by tensor based morphometry analysis were negatively correlated with high anxiety in the human intruder test. (d) Schematic of discriminative fear conditioning apparatus. The CS+ is paired with an aversive loud noise (300–600 ms car siren at 118 dB), whereas the CS− is paired with a neutral event (0.5 s of darkness). (e) Example of conditioned behavioral (vigilant scanning, time in seconds) and cardiovascular (heart rate, beats/min) responses. “Vigilant scanning” includes attentive visual search of surroundings in combination with forward extension of body/head and rearing. A conditioned heart rate rise is observed for the CS+ (blue) but not for the CS− (gray) indicating successful discrimination between the two stimuli. (f) Correlation between component 1 score (“Anxiety”) and performance in discriminative fear conditioning. High scoring in component 1 was associated with failure to achieve discriminative conditioned cardiovascular responses to CS+ and CS−. (g) Schematic representation of the marmoset *SLC6A4* promoter region showing 32 repeats. Third, fourth, and 23rd repeats containing the double and the two single‐nucleotide polymorphisms, respectively, are shaded in gray. (h) Comparison of component 1 (“Anxiety”) behavioral scores derived from the PCA of the HIT performance. Error bars represent SEM. (i) The human intruder test was used to assess anxiety levels in response to vehicle and to a single dose of 2.5 mg/kg or 10 mg/kg citalopram, 25 min prior to the intruder phase. Effects on average distance (cm) are shown (Santangelo et al., 2016). Error bars represent SEM, stars (*) represent *p* < 0.05.

A test commonly used to measure an anxious temperament in macaque monkeys is the human intruder test, whereby the experimental animal encounters an unfamiliar human (Kalin and Shelton, 1989). The unfamiliar intruder could be a potential “friend,” for instance bringing food, or they could be a threat. Hence, the ambiguity of the situation creates a state of anxiety, with subjects varying in the extent to which they approach the intruder. In the marmoset version of the task, the level of anxiety is measured based upon a component score that includes the distance the animal chooses to maintain between themselves and the intruder, their behavioral reactivity in the form of calls made, and other attentive behaviors such as body and head bobbing. This test is relatively quick to perform, as it is based in the home cage, requires no training and thus is ideal for screening large numbers of animals. Another paradigm to assess emotional reactivity in marmosets is based on the response to a snake, which is a stimulus that primates find inherently fearful [Shiba et al., 2014a, b; Fig. 3(a)]. Although the human intruder and snake test measures are somewhat independent of one another [see significant but weak correlation in Fig. 3(b)] and may measure overlapping but also distinct aspects of negative emotion, for example, innate fear versus uncertainty, they both reflect the general anxious temperament of the animal. High scores in both tests on repeated occasions indicate a high‐anxious phenotype in the marmoset. They have been linked to structural alterations in the dorsal Anterior Cingulate Cortex (dACC) [Fig. 3(c)] as measured by MRI and reductions in serotonin release in the amygdala in response to an acute dose of an SSRI, as measured by *in vivo* microdialysis (Mikheenko et al., 2015). In addition, microPET analysis of the 5‐HTT has also revealed lowered binding in the dACC related to social anxiety in marmosets (Yokoyama et al., 2013).

The translational potential of this anxiety phenotype has been determined by studying an individual marmoset's ability to display discriminative fear conditioning, and their responsivity to an uncertain or ambiguous stimulus. The tendency to generalize between threatening and non‐threatening cues and contexts, and the negative appraisal of neutral or ambiguous cues (Hirsch and Mathews, 1997; Constans et al., 1999), is characteristic of high trait‐anxious humans (Craske et al., 2009; Lissek, 2012; Dymond et al., 2015) and rats (Duvarci et al., 2009). Thus, it would be predicted that high anxious marmosets would show a similar phenotype. Consequently, Pavlovian discriminative fear conditioning has been developed for marmosets based on paradigms used effectively in humans (Lau et al., 2008; Lissek et al., 2008) and rodents (Herry et al., 2008). It involves the presentation of two distinct auditory cues, one of which is paired with a mildly aversive loud noise [300–600 ms car siren at 118 dB; the specific parameters are adjusted for each individual animal based on their performance; Fig. 3(d)]. Successful discriminative conditioning is demonstrated by the development of conditioned attentional orienting/vigilant scanning responses and heightened heart rate [Fig. 3(e)] in response to the stimulus paired with the loud noise but not the alternative stimulus paired with a neutral event, (0.5 s period of darkness). Animals that had shown higher anxiety and avoidance of the rubber snake were unable to successfully learn such a discrimination and their conditioned autonomic and behavioral responses generalized to the safety cue or to the context [Shiba et al., 2014b; Fig. 3(f)], much like high trait anxious humans who tend to generalize from threatening, aversive, and negative stimuli to neutral ones. Importantly, marmosets that failed to learn discriminative fear conditioning were perfectly successful in learning an appetitive Pavlovian discrimination paradigm by discriminating the same auditory cues when they were predictive of reward. High anxious marmosets also displayed high levels of behavioral vigilance when presented with a novel, neutral cue in a threatening context (Mikheenko et al., 2015). This is a form of pseudo‐conditioning as the aversive loud noise and a neutral stimulus were presented within the same test session but were not correlated with one another, yet high anxious marmosets still developed conditioned responses to the neutral stimulus.

High anxious marmosets and humans not only display a similar behavioral phenotype but that phenotype may also share common genetic influences. In humans, variation within the serotonin transporter gene (*SLC6A4*) that results in reduced gene expression has been linked to a high trait anxiety phenotype (Lesch et al., 1996; Canli and Lesch, 2007; Caspi et al., 2010) and marked changes in the activity of emotion circuits in the brain (Brown and Hariri, 2006; Murphy et al., 2013); although meta‐analyses have called into question the gene‐behavior association (Munafò et al., 2009; Risch et al., 2009). Recently, genetic variation in the *SLC6A4* upstream repeat region of the marmoset has also been linked to differential *SLC6A4* gene expression, with lower gene expression being associated with heightened anxiety on the human intruder test [Santangelo et al., 2016; Fig. 3(g,h)]. This gene–behavioral association in the marmoset highlights the potential advantage to studying such relationships in a purpose‐bred primate colony. The relatively controlled environment dramatically reduces the impact of variation in life experiences on an individual's behavior, allowing gene–behavior relationships to be more easily revealed compared with that of humans.

Further exploration of the phenotype has revealed that genetic variation in the *SLC6A4* gene is related to opposing effects of an acute dose of a selective serotonin reuptake inhibitor (SSRI) on anxious behavior. The low gene expressing, high trait anxious marmosets displayed an anxiogenic response in contrast to the anxiolytic response of the high gene expressing, low trait anxious marmosets [Santangelo et al., 2016; Fig. 3(i)]. These results bridge the gap between the findings in humans that report reduced responsivity to the therapeutic effects of chronic SSRI treatment in low expressing carriers with anxiety disorders (Perna et al., 2005) and depression (Keers et al., 2011; Porcelli et al., 2012), and the individual differences in sensitivity to the anxiogenic effect of acute SSRIs (Harmer and Cowen, 2013). This finding implicates *SCL6A4* genetic variation in the latter (Harmer et al., 2006; Murphy et al., 2009) which may provide insight into the underlying brain mechanisms that account for the later improvement of the clinical symptoms observed in high expressing carriers. The marmoset model therefore has enormous potential for revealing the interactions between the *SCL6A4* gene and functional activity in the serotonin system in the control of behavior.

Changes in activity of brain networks that are associated with heightened anxiety and dysregulated negative emotion, upon which serotonin may act, have been reported by functional neuroimaging studies of high trait anxious individuals in the healthy population (Killgore and Yurgelun‐Todd, 2005, Bishop, 2007) as well as in adults (Etkin and Wager, 2007; Milad and Rauch, 2007, Price & Drevets, 2010), children and adolescents (Monk et al., 2006; Guyer et al., 2008; Strawn et al., 2012) with specific anxiety disorders. These implicate a number of prefrontal subregions including the OFC and vlPFC. However, whether those changes are causal to the phenotype or merely compensatory cannot be determined in humans.

Such cause and effect has been established in marmosets, by studying the effects of selective excitotoxic lesions of the anterior OFC (antOFC) and vlPFC on fear and anxiety. At face value, the effects of both lesions seemed almost identical, increasing anxiety as measured by the human intruder test, increasing innate fear in response to a snake stimulus [Fig. 3(b)] and inducing inflexible conditioned cardiovascular and behavioral fear responses following acute alterations in the pairing of a stimulus with aversive loud noise (Agustín‐Pavón et al., 2012; Shiba et al., 2014a). This resembles the robust pattern of conditioned fear found in anxiety patients who display stronger responses to conditioned cues during extinction and are delayed in extinguishing responses compared with controls (Wessa and Flor, 2007; Duits et al., 2015). Whilst the effects of OFC lesions on tests of anxiety and innate fear have been somewhat variable in macaque monkeys (Izquierdo et al., 2005; Rudebeck et al., 2006; Kalin et al., 2007; Machado and Bachevalier, 2008) the reasons for these discrepancies have been attributed mostly to differences in lesion methodology, lesion extent and task sensitivity (for a thorough discussion on the issue see Shiba et al., 2016). The anxiety phenotype induced by antOFC and vlPFC lesions in marmosets altered the entire repertoire of behaviors that contributed to the high anxiety trait, including all aspects of behavior, coping strategy and emotionality. The finding that the lesion‐induced heightened anxiety remained within the “normal” range of such behavior displayed by marmosets within the colony [Fig. 3(b)] highlights the important role that both regions play in determining levels of trait anxiety. Moreover, the apparently similar symptomatology associated with lesions to two distinct regions of PFC illustrates the multivariate nature of anxiety.

The question remains as to what is the distinct contribution of these two prefrontal regions to the anxiety phenotype, the answer of which will help to stratify anxiety disorders and develop targeted treatments. Besides the failure to regulate negative emotions, patients with anxiety and depression often make poor decisions. Because they are particularly sensitive to negative information, they tend to perceive threat with increased intensity and are more risk averse (Murphy et al., 2003; Dickson, 2006; Smoski et al., 2008; Mueller et al., 2010) and thus display negative biases when making decisions (Bradley et al., 1998; Murphy et al., 1999; Mogg et al., 2000; Williams et al., 2007; Peckham et al., 2010). Indeed, reductions in serotonin within the OFC of marmosets have been implicated in such negative biases (Rygula et al., 2015) as identified in a probabilistic discrimination task similar to that used to reveal altered activity in prefronto‐amygdala circuitry related to negative biases in depressed patients (Taylor Tavares et al., 2008).

To compare the impact of antOFC and vlPFC inactivation in marmosets on decision making we developed an approach‐avoidance instrumental decision‐making task. In this task, animals optimize their reward by responding equally to two, equivalently rewarded left and right locations on a touch‐sensitive computer screen. In occasional probe sessions, superimposed over the reward schedule, an aversive loud noise, a form of punishment, is associated with responding at one of the two locations. This results in a conflict at that location between responding to gain reward, and avoiding responding due to the punishment. However, animals normally maintain responding to both stimuli, choosing to optimize reward, despite the occasional punishment. However, transient pharmacological inactivation of the antOFC and vlPFC alters performance in distinct ways. Inactivation of the vlPFC has no impact on overall levels of responding but causes the animal to avoid making the punished response, resulting in many more responses being made to the side on which responding only receives reward. Hence, the cost‐benefit balance of responding for reward in the face of punishment is switched from a positive approach response to a negative avoidance response. In contrast, inactivation of antOFC has no effect on the day of receiving punishment, but it acts to bias responding away from the punished side the following day, presumably as a consequence of its effects on memory consolidation (Clarke et al., 2015).

The vlPFC has been implicated in attentional processing and specifically the shifting of higher‐order attentional sets as described in the cognitive section above. Thus, a parsimonious account of the on‐line negative bias induced by inactivation of the vlPFC is that the marmoset is unable to shift attention away from the intrinsically salient aversive loud noise and toward the rewarding aspects of the context in order to perform a cost‐benefit analysis. Consequently, this prolonged attention to the aversive stimulus leads to the negative bias. This hypothesis is also consistent with the contribution of the vlPFC to cognitive re‐appraisal, whereby individuals explicitly shift their attention from a salient negative interpretation of a context to a more positive interpretation (Buhle et al., 2014), and down‐regulation of emotion during conflict and acceptance of unfair outcomes (Tabibnia et al., 2008; Feng et al., 2015). Consequently, it is proposed that a compromised vlPFC induces anxiety because subjects fail to switch their attention away from negative events.

On the other hand, the antOFC inactivation‐induced negative bias is not linked to the online appraisal and processing of the negative stimulus but rather to its memory. The OFC has been linked to the development of stimulus‐reward associations and the ability to predict outcomes, particularly within probabilistic contexts (Schoenbaum and Roesch, 2005; Schoenbaum et al., 2007; Balleine et al., 2011; Walton et al., 2011; Rudebeck and Murray, 2014). Therefore, it can be argued that a compromised OFC makes the punishment no longer predictable. This learned state of uncertainty leads to establishment of a stronger punishment memory that underlies the negative bias and avoidance behavior upon its retrieval the next day. This is consistent with the role of uncertain environments in the development of anxious responses (Grupe and Nitschke, 2013). The negative bias is abolished by inactivation of either the amygdala or the hippocampus on the day of retrieval, or disconnection of the two, implicating an OFC‐amygdala‐hippocampal network that drives the expression of such negative biases. This account of the symptoms of anxiety and depression induced by compromised OFC function differs from that proposed above for vlPFC dysfunction. Instead of an inability to down‐regulate responses on‐line, a dysfunctional OFC may leave the system unregulated and result in the learning of maladaptive negative associations due to a failure to predict aversive outcomes, supporting the negative cognitions prevalent in anxiety and depression.

In summary, the marmoset model has allowed the investigation of regulation and dysregulation within the negative valence system (Fig. 1) at both the individual‐ (trait anxiety and genetic variation in the serotonin transporter gene) and circuit‐based levels (prefronto‐amygdala‐hippocampal manipulations). The finding of an interaction between the serotonin transporter gene and the effects of acute SSRI treatment on anxious behavior has implications for the individual targeting of treatments. On the other hand, the proposed roles of vlPFC and antOFC, respectively, in attentional processing and in the prediction of negative outcomes, provide an important framework for the stratification of anxiety disorders. Future studies will focus on the contributions of other regions of PFC that have been implicated in the regulation of negative emotion, including primate dorsolateral PFC (Buhle et al., 2014) and peri‐ and sub‐genual anterior cingulate cortex (Drevets et al., 2008; Etkin et al., 2011). While numerous studies have implicated the prelimbic and infralimbic regions of ACC in rodents, respectively, in the expression and extinction of freezing to a conditioned fear stimulus, their putative homology to primate peri‐and subgenual ACC remains unclear (for a critical review see Myers‐Schulz and Koenigs, 2012). This further emphasizes the importance of the marmoset primate model in parsing out the distinct cognitive functions of, and interactions between, these prefrontal brain regions and their downstream targets, in the control of negative emotion. It will also provide the basis by which we can determine the neurobiological and psychological mechanisms underlying the action of current pharmacotherapies that target the serotonin (e.g., SSRIs), noradrenaline (selective noradrenergic reuptake inhibitors) and glutamatergic (e.g., ketamine) systems in the treatment of dysregulated emotions. Only then can these treatments be targeted appropriately to the individual.

## IMPAIRED POSITIVE VALENCE: MODELING ANHEDONIA

Positive valence systems, as defined by the RDoC framework, are responsible for responding to positive situations and contexts, including reward seeking, consummatory behaviors and reward/habit learning. Dysfunction within these systems occurs in a number of major psychiatric disorders. One specific example of a clinical symptom involving disturbed positive valence is anhedonia (Fig. 1). Anhedonia is defined according to the DSM (I–V) as “decreased interest or pleasure in most activities, most of each day” and is prevalent in about 37% of depressed patients (Kessler et al., 2009). Indeed, for a patient to be diagnosed with depression they must suffer from one or other form of anhedonia or depressed mood (DSM V). Besides depression, anhedonia constitutes one of the primary negative symptoms of schizophrenia, being present in half of the patients (Pelizza and Ferrari, 2009), is present in eating disorders (Davis and Woodside, 2002) and is associated with withdrawal in drug abuse (Markou and Koob, 1991). It has also been reported in a number of neurodegenerative disorders, including Parkinson's Disease (Isella et al., 2003) and Alzheimer's disease (Starkstein et al., 2005).

Clinically, it has been identified by self‐report measures of hedonic experiences using a variety of questionnaires, such as the Snaith Hamilton (Snaith et al., 1995) and Fawcett–Clark Pleasure scales (Fawcett et al., 1983). The majority of the questions on these scales focus on the consummatory aspects of positive experiences such as “one food tastes as good as another to me.” As a consequence, pre‐clinical models of depression have tended to use the sucrose consumption test as the primary measure of anhedonia (Slattery et al., 2007) when investigating novel pharmacotherapies. However, preclinical neurobiological studies of positive valence systems have recognized motivational, reinforcing, decision making and consummatory components [see Treadway and Zald (2011) for a comprehensive review], all of which can be parsed at the level of neural circuits and neurochemistry. Thus, anhedonia could arise from a lack of the ability not only to experience pleasure (consummatory anhedonia) but also to anticipate pleasure (anticipatory anhedonia). Indeed, a failure to anticipate pleasure has a major impact on an individual's motivation to seek out pleasure (motivational anhedonia). Treadway and Zald (2011) further proposed the concept of decisional anhedonia to include the effects of anhedonia on decision making in the context of reward.

Recently, distinctions between these different types of anhedonia have begun to emerge in clinical research. For example, patients with depression show anticipatory (McFarland and Klein, 2009) as opposed to consummatory anhedonia (Dichter et al., 2010). On the other hand, schizophrenia patients show intact anticipation of reward but deficits in motivational/effortful responding for reward, a form of apathy (Gard et al., 2009, 2014). Another distinction to have arisen is that between self‐report measures of pleasurable experiences retrospectively and experiencing pleasure per se. In particular, patients with schizophrenia and depression do not show reductions in their hedonic response to different sucrose solutions compared with controls, despite scoring high on the Chapman and Fawcett anhedonia questionnaires (Amsterdam et al., 1987; Berlin et al., 1998). Moreover, self‐reported affective flattening and anhedonia in schizophrenia, as measured by questionnaires, is actually associated with an almost normal experience of emotion as measured by hedonic ratings during the experience of pleasant stimuli, including pictures, films, words, or faces (Burbridge and Barch, 2007; Heerey and Gold, 2007; Trémeau et al., 2010). This inconsistency between retrospective self‐reports of anhedonia and the actual on‐line experience may well be a consequence of the reliance of clinical questionnaires on the ability of patients to “represent” hedonic experience, as opposed to the hedonic experience per se. This ability to “represent” consummatory pleasure is also a major component of anticipatory and motivational hedonic processes and thus illustrates the difficulty in interpreting self‐report questionnaires in the clinical population.

Considerable insight into the distinct consummatory, anticipatory and motivational components of reward processing has been gained from neurobiological and neurochemical studies at the level of the striatum and amygdala of both rodents (Cardinal et al., 2002; Wassum and Izquierdo, 2015) and monkeys (Schultz et al., 2000; Schultz, 2004; Murray, 2007; Bermudez and Schultz, 2010). The anhedonia associated with depression, however, is linked to altered activity within the ventromedial PFC (Keedwell et al., 2005). This is a complex region in humans composed of a number of discrete cytoarchitectonic areas, including Brodmann's areas 10, 14, 25, and 32, and their contribution to the regulation of reward processing and their interaction with striatal and amygdala reward systems is unknown. This again highlights the need for studies in a primate in which the organization of ventromedial PFC appears far similar to humans than that of rodents (Myers‐Schulz and Koenigs, 2012).

**Figure 4:**
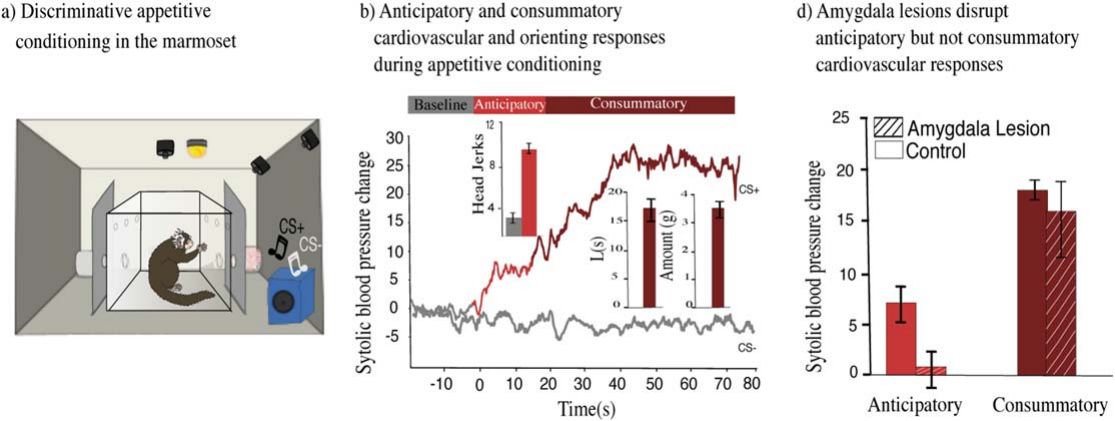
Parsing consummatory and anticipatory aspects of positive valence in the marmoset. (a) Schematic of discriminative appetitive conditioning apparatus. CS+ resulted in opening of the door revealing food box with reward (e.g., right), whereas CS− resulted in presentation of empty food box (e.g., left). The anticipatory period entails viewing the full food box and is followed by the consummatory period, when marmosets are allowed access to the reward. (b) Conditioned behavioral response during the anticipatory phase is measured by the number of orienting head movements (head jerks) toward the food box. Consummatory behavior is measured by the latency to eat the reward (“L”, seconds) and the amount of reward eaten (grams). A conditioned systolic blood pressure rise is observed for the CS+ (Light red for anticipatory period, dark red for consummatory period) but not for the CS− (gray). (c) Systolic blood pressure response during the CS+ after excitotoxic lesions of the amygdala (lined bars) presented for the anticipatory (Light red) and the consummatory (Dark red) periods compared with controls (empty bars) (Braesicke et al., 2005). Error bars represent SEM, stars (*) represent *p* < 0.05.

We have developed a paradigm in the marmoset that distinguishes between the consummatory and anticipatory aspects of pleasure and uses independent measures of the behavioral and autonomic arousal (cardiovascular) components. The latter is seldom measured in studies of reward processing in animals (but see Rudebeck et al., 2014) but can be an important component of positive emotional responses, as it is for negative emotions, and is often the metric used in humans, alongside self‐report questionnaires. Using a Pavlovian procedure [Fig. 4(a)], one version of the paradigm used the “sight of the food” viewed through a transparent door of a food box as the conditioned stimulus. This constitutes the anticipatory period and was followed by the consummatory period when the door opened, resulting in access to the reward. With training, a conditioned rise in systolic blood pressure accompanied by approach behavior toward the food box developed during the anticipatory period, as the animals learned that the presentation of the food box was predictive of subsequent reward [Fig. 4(b)]. In contrast, the rise in blood pressure that accompanied the consummatory period was present from the beginning. The consummatory arousal was, nevertheless, motivational in nature since it was only seen in the context of the marmosets gaining access to their preferred, high incentive reward and not the food pellets that they received daily (Braesicke et al., 2005). Consistent with the role of the amygdala in appetitive conditioning, excitotoxic lesions of the amygdala in the marmoset reduced anticipatory behavioral responses directed toward the conditioned stimulus, as shown previously in rodents (Holland, 1999) and also reduced the conditioned cardiovascular arousal. In contrast there was no effect on the food box approach response or the cardiovascular arousal during the consummatory period. Thus, damage to the amygdala induced anticipatory but not consummatory anhedonia [Fig. 4(c)].

A revised version of the Pavlovian paradigm has since been developed to identify the brain mechanisms underlying the ability of marmosets to adapt their anticipatory hedonic responses to changes in environmental contingencies. Animals were conditioned to discriminate between two auditory stimuli, either predicting reward (CS+) or an empty food box (CS−). Changes in behavioral and cardiovascular arousal during the CSs were assessed both after a single extinction session, in which the decline in conditioned cardiovascular arousal was measured following omission of the expected reward and after reversal of the reinforcing contingencies, in which the previous CS− became the CS+, and vice versa. So far this revised version has been used to study the contribution of the antOFC to the regulation of appetitive Pavlovian conditioning. Marmosets with excitotoxic lesions of the antOFC failed to show the normal decline in the conditioned systolic blood pressure response after reward omission, instead showing a prolonged arousal response. Moreover, upon reversal, antOFC lesioned animals were much slower to reverse their blood pressure response. Significantly, even when the cardiovascular response was successfully reversed, conditioned behavior was not (Reekie et al., 2008). This was in marked contrast to controls in which there was strong coupling between the rate of reversal learning of the conditioned behavioral and cardiovascular responses. Thus, antOFC lesions caused uncoupling of cardiovascular and behavioral responses. This is particularly relevant for the motivational‐anticipatory distinction in anhedonia, as a patient may display cardiovascular anticipatory arousal, but due to uncoupling this may fail to result in motivated behavior.

Future studies in marmosets are ideally placed to focus on the contribution of the distinct regions of the ventromedial PFC in the regulation of striatal and amygdala reward processing, including their modulation of the monoamine systems. Downregulation of striatal activity (Heller et al., 2009; Robinson et al., 2012) and of striatal dopamine receptors in particular (Cannon et al., 2009), has been reported in depressed subjects, along with reductions in amygdala responsivity to happy faces (Beesdo et al., 2009), the latter being correlated with symptoms of anhedonia (Stuhrmann et al., 2013). An understanding of the relationship between these cortical and subcortical changes and their causal role in the anhedonic symptoms will inform treatment strategies since the symptom of anhedonia appears particularly resistant to current pharmacotherapies. It is largely unresponsive to SSRI based treatments of depression (Shelton and Tomarken, 2001; Dunlop and Nemeroff, 2007) and similarly fails to improve when SSRI administration is used as an add‐on therapy in schizophrenia (Sepehry et al., 2007). Whilst drugs that target the dopamine system are successful in alleviating many symptoms of depression and schizophrenia, reports of the direct effects of such drugs on anhedonic symptoms are mixed (Nutt et al., 2007). This may not be surprising since evidence from psychopharmacological experiments in animals implicates dopamine primarily in motivational and reward prediction processes rather than pleasure per se (Robbins and Everitt, 1992; Salamone, 1997; Berridge and Robinson, 1998; Denk et al., 2005; Schultz et al., 2015). Thus, depending upon what type of anhedonia a patient is displaying, for example, consummatory, anticipatory, or motivational, the neurochemical target for alleviating anhedonia may differ.

## UTILITY OF THE MARMOSET IN FUTURE INVESTIGATIONS OF NEURAL AND COGNITIVE DEVELOPMENT RELEVANT TO MENTAL HEALTH

Seventy five per cent of patients with a mental disorder will have shown neuropsychiatric symptoms by age 25 (Kessler et al., 2005), making the first years of life extremely important for understanding the development of neuropsychiatric disorders and their behavioral dimensions. The marmoset has distinct but, relatively, short duration developmental stages, compared with old world monkeys, lasting just a few months each, until they reach adulthood and sexual maturity at approximately 18‐20 months (Abbott et al., 2003; see Schultz‐Darken et al., 2016 for a comprehensive review). These developmental stages have been well characterized in terms of typical behaviors observed, including social interactions (Chalmers and Locke‐Haydon, 1984), vocalizations (Pistorio et al., 2006; Braun et al., 2015), and sensorimotor abilities (Piper et al., 1992; Kaplan and Rogers, 2006; Izumi et al., 2012). Moreover, the impact of early life experiences, such as maternal deprivation and infant isolation, on some of these behavioral outcomes, as well as on levels of cortisol, adrenaline and noradrenaline are already known (Dettling et al., 2002; Pryce et al., 2004; Dettling et al., 2007). This research provides the community with a useful toolbox to study gene‐environment interactions and the effects of early life experiences on cognitive and affective development and pinpoints the different milestones of physical ability according to age. As Schultz‐Darken et al. (2016) highlight, marmosets pose the great advantage of primarily breeding twins, which allows the direct comparison of subjects with the same genetic background after being placed in different experimental conditions. What remains to be studied is how cognitive functions are acquired in the marmoset in relation to brain development in order to inform our understanding of the emergence of neuropsychiatric disorders.

In humans, the development of cognitive functions, including reversal learning and attentional set‐shifting occur at different ages (Luciana, 2003; Davidson et al., 2006) and are affected by early life experiences and the home environment (Sarsour et al., 2011). Unraveling the genetic and environmental contribution to this development is difficult in humans because of the complexity of, and the lack of experimental control over, their environment. In the marmoset, the relationship between the development of these cognitive processes and their underlying neural circuit can be determined in a highly controlled environment. Taking advantage of their quick progression from infancy to adulthood, the common problem of high attrition rates in human studies of development (Barnett, 1995) and the impossibility of following subjects over a period of twenty years can be overcome. Young marmosets can be assessed with CANTAB tasks using a home‐cage apparatus that allows testing in a relaxed environment (Crofts et al., 1999; Takemoto et al., 2011) while neuroimaging will be used to determine brain alterations across the different developmental stages. These aspects of development can also be studied as predictors of anxious phenotypes in adulthood in conjunction with genetic variations, which would provide a rich dataset for studying the development of neuropsychiatric disorders.

## CONCLUSIONS

In conclusion, the marmoset is proving a valuable species in which to study many of the symptoms of neuropsychiatric disorders associated with dysfunction within cognitive, negative and positive valence systems (Fig. 1). In the marmoset, a range of tests have been developed which are designed to dissect out the behavioral dimensions that underlie these symptoms. Some of these dimensions are already being successfully mapped on to specific prefrontal circuits and neurochemical pathways within the marmoset using a range of neurobiological techniques including temporary or permanent brain manipulations, microdialysis, microPET, and structural MRI. The new generation of viral mediated tools for targeting chemically specific neural pathways, including opto‐ and pharmacogenetics, in combination with fluorodeoxyglucose and receptor ligand based microPET and high field MRI for measuring functional and resting state activity will reveal interactions between and within cognitive and emotional circuits. While marmosets may not replace Old World macaques for studying certain aspects of higher‐order executive functioning, their compact, but highly developed primate brains, small body size and thus ease of keeping large groups of marmosets in spacious accommodation make them the ideal primate for large scale research programs investigating cortical–subcortical circuit interactions. Moreover, their relatively short 5 month gestation and 2 year period of development provides a major opportunity to determine the effects of genetic and behavioral risk factors for neuropsychiatric disorders on the development of these neurocognitive circuits and neurochemical modulatory pathways across childhood and adolescence, in order to understand their impact on complex cognitive and emotional behaviors.

This work was supported by long term funding from the MRC (ACR) and the Wellcome Trust (TWR) and was performed within the Behavioral and Clinical Neuroscience Institute, University of Cambridge, funded jointly by the Wellcome Trust and MRC. LO is supported by an MRC studentship. We thank Stacey Jackson, Ian Bolton and Adrian Newman for advice on illustration and figure formatting.

## References

[dneu22446-bib-0001] Abbott DH , Hearn JP. 1978 Physical, hormonal and behavioural aspects of sexual development in the marmoset monkey, Callithrix jacchus. Reproduction 53:155–166. 10.1530/jrf.0.0530155 417178

[dneu22446-bib-0002] Abbott DH , Barnett DK , Colman RJ , Yamamoto ME , Schultz‐Darken NJ. 2003 Aspects of common marmoset basic biology and life history important for biomedical research. Comp Med 53:339–350. 14524409

[dneu22446-bib-0003] Abbruzzese M , Ferri S , Scarone S. 1995 Wisconsin Card Sorting Test performance in obsessive‐compulsive disorder: No evidence for involvement of dorsolateral prefrontal cortex. Psychiatry Res 58:37–43. 8539310 10.1016/0165-1781(95)02670-r

[dneu22446-bib-0004] Abbruzzese M , Ferri S , Scarone S. 1997 The selective breakdown of frontal functions in patients with obsessive–compulsive disorder and in patients with schizophrenia: A double dissociation experimental finding. Neuropsychologia 35:907–912. 9204494 10.1016/s0028-3932(96)00095-4

[dneu22446-bib-0005] Agustín‐Pavón C , Braesicke K , Shiba Y , Santangelo AM , Mikheenko Y , Cockroft G , Asma F , Clarke H , Man M‐S , Roberts AC. 2012 Lesions of ventrolateral prefrontal or anterior orbitofrontal cortex in primates heighten negative emotion. Biol Psychiatry 72:266–272. 22502990 10.1016/j.biopsych.2012.03.007

[dneu22446-bib-0006] Airaksinen E , Larsson M , Forsell Y. 2005 Neuropsychological functions in anxiety disorders in population‐based samples: Evidence of episodic memory dysfunction. J Psychiatr Res 39:207–214. 15589570 10.1016/j.jpsychires.2004.06.001

[dneu22446-bib-0007] Amsterdam JD , Settle RG , Doty RL , Abelman E , Winokur A. 1987 Taste and smell perception in depression. Biol Psychiatry 22:1481–1485. 3676376 10.1016/0006-3223(87)90108-9

[dneu22446-bib-0008] Arai S , Okamoto Y , Fujioka T , Inohara K , Ishitobi M , Matsumura Y , Jung M , Kawamura K , Takiguchi S , Tomoda A , et al. 2015 Altered frontal pole development affects self‐generated spatial working memory in ADHD. Brain Dev 38:471–480. 26709204 10.1016/j.braindev.2015.11.005

[dneu22446-bib-0009] Arnaudova I , Krypotos A‐M , Effting M , Boddez Y , Kindt M , Beckers T. 2013 Individual differences in discriminatory fear learning under conditions of ambiguity: A vulnerability factor for anxiety disorders?. Front Psychol 4:298. 23755030 10.3389/fpsyg.2013.00298 PMC3664781

[dneu22446-bib-0010] Arnsten AFT , Goldman‐Rakic PS. 1984 Selective prefrontal cortical projections to the region of the locus coeruleus and raphe nuclei in the rhesus monkey. Brain Res 306:9–18. 6466989 10.1016/0006-8993(84)90351-2

[dneu22446-bib-0011] Arroll B , Macgillivray S , Ogston S , Reid I , Sullivan F , Williams B , Crombie I. 2005 Efficacy and tolerability of tricyclic antidepressants and SSRIs compared with placebo for treatment of depression in primary care: A meta‐analysis. Ann Fam Med 3:449–456. 16189062 10.1370/afm.349 PMC1466912

[dneu22446-bib-0012] Badcock JC , Michie PT , Rock D. 2005 Spatial working memoryand planning ability: Contrasts between schizophreniaand bipolar i disorder. Cortex 41:753–763. 16350658 10.1016/s0010-9452(08)70294-6

[dneu22446-bib-0013] Balleine BW , Leung BK , Ostlund SB. 2011 The orbitofrontal cortex, predicted value, and choice. Ann N Y Acad Sci 1239:43–50. 22145874 10.1111/j.1749-6632.2011.06270.x

[dneu22446-bib-0014] Bar‐Haim Y , Lamy D , Pergamin L , Bakermans‐Kranenburg MJ , van IJzendoorn MH. 2007 Threat‐related attentional bias in anxious and nonanxious individuals: A meta‐analytic study. Psychol Bull 133:1–24. 17201568 10.1037/0033-2909.133.1.1

[dneu22446-bib-0015] Barnett WS. 1995 Long‐term effects of early childhood programs on cognitive and school outcomes. Futur Child 5:25. 8835514

[dneu22446-bib-0016] Beatty WW , Monson N. 1990 Problem solving in parkinson's disease: Comparison of performance on the wisconsin and california card sorting tests. J Geriatr Psychiatry Neurol 3:163–171. 2282133 10.1177/089198879000300308

[dneu22446-bib-0017] Bedard A‐C , Martinussen R , Ickowicz A , Tannock R. 2004 Methylphenidate improves visual‐spatial memory in children with attention‐deficit/hyperactivity disorder. J Am Acad Child Adolesc Psychiatry 43:260–268. 15076258 10.1097/00004583-200403000-00006

[dneu22446-bib-0018] Beesdo K , Lau JYF , Guyer AE , McClure‐Tone EB , Monk CS , Nelson EE , Fromm SJ , Goldwin MA , Wittchen H‐U , Leibenluft E , et al. 2009 Common and distinct amygdala‐function perturbations in depressed vs anxious adolescents. Arch Gen Psychiatry 66:275–285. 19255377 10.1001/archgenpsychiatry.2008.545 PMC2891508

[dneu22446-bib-0020] Belzung C , Griebel G. 2001 Measuring normal and pathological anxiety‐like behaviour in mice: A review. Behav Brain Res 125:141–149. 11682105 10.1016/s0166-4328(01)00291-1

[dneu22446-bib-0021] Berg EA. 1948 A simple objective technique for measuring flexibility in thinking. J Gen Psychol 39:15–22. 18889466 10.1080/00221309.1948.9918159

[dneu22446-bib-0022] Berlin I , Givry‐Steiner L , Lecrubier Y , Puech AJ. 1998 Measures of anhedonia and hedonic responses to sucrose in depressive and schizophrenic patients in comparison with healthy subjects. Eur Psychiatry 13:303–309. 19698645 10.1016/S0924-9338(98)80048-5

[dneu22446-bib-0023] Bermudez MA , Schultz W. 2010 Reward magnitude coding in primate amygdala neurons. J Neurophysiol 104:3424–3432. 20861431 10.1152/jn.00540.2010 PMC3007636

[dneu22446-bib-0024] Berridge KC , Robinson TE. 1998 What is the role of dopamine in reward: Hedonic impact, reward learning, or incentive salience?. Brain Res Rev 28:309–369. 9858756 10.1016/s0165-0173(98)00019-8

[dneu22446-bib-0025] Birrell JM , Brown VJ. 2000 Medial frontal cortex mediates perceptual attentional set shifting in the rat. J Neurosci 20:4320–4324. 10818167 10.1523/JNEUROSCI.20-11-04320.2000 PMC6772641

[dneu22446-bib-0726] Bishop SJ . 2007 Neurocognitive mechanisms of anxiety: an integrative account. Trends Cogn Sci 11:307–316. 17553730 10.1016/j.tics.2007.05.008

[dneu22446-bib-0026] Bissonette GB , Martins GJ , Franz TM , Harper ES , Schoenbaum G , Powell EM. 2008 Double dissociation of the effects of medial and orbital prefrontal cortical lesions on attentional and affective shifts in mice. J Neurosci 28:11124–11130. 18971455 10.1523/JNEUROSCI.2820-08.2008 PMC2657142

[dneu22446-bib-0027] Bradley BP , Mogg K , Falla SJ , Hamilton LR. 1998 Attentional bias for threatening facial expressions in anxiety: Manipulation of stimulus duration. Cogn Emot 12:737–753.

[dneu22446-bib-0028] Braesicke K , Parkinson JA , Reekie Y , Man M‐S , Hopewell L , Pears A , Crofts H , Schnell CR , Roberts AC. 2005 Autonomic arousal in an appetitive context in primates: A behavioural and neural analysis. Eur J Neurosci 21:1733–1740. 15845101 10.1111/j.1460-9568.2005.03987.x

[dneu22446-bib-0029] Braun K , Schultz‐Darken N , Schneider M , Moore CF , Emborg ME. 2015 Development of a novel postnatal neurobehavioral scale for evaluation of common marmoset monkeys. Am J Primatol 77:401–417. 25676438 10.1002/ajp.22356 PMC4374045

[dneu22446-bib-0030] Brown SM , Hariri AR. 2006 Neuroimaging studies of serotonin gene polymorphisms: Exploring the interplay of genes, brain, and behavior. Cogn Affect Behav Neurosci 6:44–52. 16869228 10.3758/cabn.6.1.44

[dneu22446-bib-0031] Buchy L , Hawco C , Joober R , Malla A , Lepage M. 2015 Cognitive insight in first‐episode schizophrenia: Further evidence for a role of the ventrolateral prefrontal cortex. Schizophr Res 166:65–68. 26004692 10.1016/j.schres.2015.05.009

[dneu22446-bib-0032] Buhle JT , Silvers JA , Wager TD , Lopez R , Onyemekwu C , Kober H , Weber J , Ochsner KN. 2014 Cognitive reappraisal of emotion: A meta‐analysis of human neuroimaging studies. Cereb Cortex 24:2981–2990. 23765157 10.1093/cercor/bht154 PMC4193464

[dneu22446-bib-0033] Burbridge JA , Barch DM. 2007 Anhedonia and the experience of emotion in individuals with schizophrenia. J Abnorm Psychol 116:30–42. 17324014 10.1037/0021-843X.116.1.30

[dneu22446-bib-0034] Burman KJ , Rosa MGP. 2009 Architectural subdivisions of medial and orbital frontal cortices in the marmoset monkey (Callithrix jacchus). J Comp Neurol 514:11–29. 19260047 10.1002/cne.21976

[dneu22446-bib-0035] Burman KJ , Palmer SM , Gamberini M , Rosa MGP. 2006 Cytoarchitectonic subdivisions of the dorsolateral frontal cortex of the marmoset monkey (Callithrix jacchus), and their projections to dorsal visual areas. J Comp Neurol 495:149–172. 16435289 10.1002/cne.20837

[dneu22446-bib-0036] Cañas J , Quesada JF , Antolí A , Fajardo I. 2003 Cognitive flexibility and adaptability to environmental changes in dynamic complex problem‐solving tasks. Ergonomics 46:482–501. 12745698 10.1080/0014013031000061640

[dneu22446-bib-0037] Canavan AGM , Passingham RE , Marsden CD , Quinn N , Wyke M , Polkey CE. 1989 The performance on learning tasks of patients in the early stages of Parkinson's disease. Neuropsychologia 27:141–156. 2927625 10.1016/0028-3932(89)90167-x

[dneu22446-bib-0038] Canli T , Lesch K‐P. 2007 Long story short: The serotonin transporter in emotion regulation and social cognition. Nat Neurosci 10:1103–1109. 17726476 10.1038/nn1964

[dneu22446-bib-0039] Cannon DM , Klaver JM , Peck SA , Rallis‐Voak D , Erickson K , Drevets WC. 2009 Dopamine type‐1 receptor binding in major depressive disorder assessed using positron emission tomography and [11C]NNC‐112. Neuropsychopharmacology 34:1277–1287. 18946469 10.1038/npp.2008.194 PMC2656589

[dneu22446-bib-0040] Cardinal RN , Parkinson JA , Hall J , Everitt BJ. 2002 Emotion and motivation: The role of the amygdala, ventral striatum, and prefrontal cortex. Neurosci Biobehav Rev 26:321–352. 12034134 10.1016/s0149-7634(02)00007-6

[dneu22446-bib-0041] Carmichael ST , Price JL. 1996 Connectional networks within the orbital and medial prefrontal cortex of macaque monkeys. J Comp Neurol 371:179–207. 8835726 10.1002/(SICI)1096-9861(19960722)371:2<179::AID-CNE1>3.0.CO;2-#

[dneu22446-bib-0042] Caspi A , Hariri AR , Holmes A , Uher R , Moffitt TE. 2010 Genetic sensitivity to the environment: The case of the serotonin transporter gene and its implications for studying complex diseases and traits. Focus (Madison) 8:398–416. 10.1176/appi.ajp.2010.09101452 PMC2943341 20231323

[dneu22446-bib-0043] Castellanos FX , Sonuga‐Barke EJS , Milham MP , Tannock R. 2006 Characterizing cognition in ADHD: Beyond executive dysfunction. Trends Cogn Sci 10:117–123. 16460990 10.1016/j.tics.2006.01.011

[dneu22446-bib-0044] Chalmers NR , Locke‐Haydon J. 1984 Correlations among measures of playfulness and skillfulness in captive common marmosets (Callithrix jacchus jacchus). Dev Psychobiol 17:191–208. 6423428 10.1002/dev.420170209

[dneu22446-bib-0045] Chamberlain SR , Blackwell AD , Fineberg NA , Robbins TW , Sahakian BJ. 2005 The neuropsychology of obsessive compulsive disorder: The importance of failures in cognitive and behavioural inhibition as candidate endophenotypic markers. Neurosci Biobehav Rev 29:399–419. 15820546 10.1016/j.neubiorev.2004.11.006

[dneu22446-bib-0046] Chamberlain SR , Fineberg NA , Blackwell AD , Robbins TW , Sahakian BJ. 2006 Motor inhibition and cognitive flexibility in obsessive‐compulsive disorder and trichotillomania. Am J Psychiatry 163:1282–1284. 16816237 10.1176/ajp.2006.163.7.1282

[dneu22446-bib-0047] Chamberlain SR , Fineberg NA , Blackwell AD , Clark L , Robbins TW , Sahakian BJ. 2007 A neuropsychological comparison of obsessive‐compulsive disorder and trichotillomania. Neuropsychologia 45:654–662. 17005210 10.1016/j.neuropsychologia.2006.07.016

[dneu22446-bib-0048] Chamberlain SR , Menzies L , Hampshire A , Suckling J , Fineberg NA , del Campo N , Aitken M , Craig K , Owen AM , Bullmore ET , et al. 2008 Orbitofrontal dysfunction in patients with obsessive‐compulsive disorder and their unaffected relatives. Science 321:421–422. 18635808 10.1126/science.1154433

[dneu22446-bib-0049] Chase HW , Clark L , Sahakian BJ , Bullmore ET , Robbins TW. 2008 Dissociable roles of prefrontal subregions in self‐ordered working memory performance. Neuropsychologia 46:2650–2661. 18556028 10.1016/j.neuropsychologia.2008.04.021

[dneu22446-bib-0050] Clark L , Blackwell AD , Aron AR , Turner DC , Dowson J , Robbins TW , Sahakian BJ. 2007 Association between response inhibition and working memory in adult ADHD: A link to right frontal cortex pathology?. Biol Psychiatry 61:1395–1401. 17046725 10.1016/j.biopsych.2006.07.020

[dneu22446-bib-0051] Clarke HF , Dalley JW , Crofts HS , Robbins TW , Roberts AC. 2004 Cognitive inflexibility after prefrontal serotonin depletion. Science 304:878–880. 15131308 10.1126/science.1094987

[dneu22446-bib-0052] Clarke HF , Walker SC , Crofts HS , Dalley JW , Robbins TW , Roberts AC. 2005 Prefrontal serotonin depletion affects reversal learning but not attentional set shifting. J Neurosci 25:532–538. 15647499 10.1523/JNEUROSCI.3690-04.2005 PMC6725478

[dneu22446-bib-0053] Clarke HF , Walker SC , Dalley JW , Robbins TW , Roberts AC. 2007 Cognitive inflexibility after prefrontal serotonin depletion is behaviorally and neurochemically specific. Cereb Cortex 17:18–27. 16481566 10.1093/cercor/bhj120

[dneu22446-bib-0054] Clarke HF , Robbins TW , Roberts AC. 2008 Lesions of the medial striatum in monkeys produce perseverative impairments during reversal learning similar to those produced by lesions of the orbitofrontal cortex. J Neurosci 28:10972–10982. 18945905 10.1523/JNEUROSCI.1521-08.2008 PMC3981993

[dneu22446-bib-0055] Clarke HF , Hill GJ , Robbins TW , Roberts AC. 2011 Dopamine, but not serotonin, regulates reversal learning in the marmoset caudate nucleus. J Neurosci 31:4290–4297. 21411670 10.1523/JNEUROSCI.5066-10.2011 PMC3083841

[dneu22446-bib-0056] Clarke HF , Horst NK , Roberts AC. 2015 Regional inactivations of primate ventral prefrontal cortex reveal two distinct mechanisms underlying negative bias in decision making. Proc Natl Acad Sci U S A 112:4176–4181. 25775597 10.1073/pnas.1422440112 PMC4386364

[dneu22446-bib-0057] Clatworthy PL , Lewis SJG , Brichard L , Hong YT , Izquierdo D , Clark L , Cools R , Aigbirhio FI , Baron J‐C , Fryer TD , et al. 2009 Dopamine release in dissociable striatal subregions predicts the different effects of oral methylphenidate on reversal learning and spatial working memory. J Neurosci 29:4690–4696. 19369539 10.1523/JNEUROSCI.3266-08.2009 PMC6665353

[dneu22446-bib-0058] Collins P , Roberts AC , Dias R , Everitt BJ , Robbins TW. 1998 Perseveration and strategy in a novel spatial self‐ordered sequencing task for nonhuman primates: Effects of excitotoxic lesions and dopamine depletions of the prefrontal cortex. J Cogn Neurosci 10:332–354. 9869708 10.1162/089892998562771

[dneu22446-bib-0059] Constans JI , Penn DL , Ihen GH , Hope DA. 1999 Interpretive biases for ambiguous stimuli in social anxiety. Behav Res Ther 37:643–651. 10402689 10.1016/s0005-7967(98)00180-6

[dneu22446-bib-0060] Craske MG , Rauch SL , Ursano R , Prenoveau J , Pine DS , Zinbarg RE. 2009 What is an anxiety disorder?. Depress Anxiety 26:1066–1085. 19957279 10.1002/da.20633

[dneu22446-bib-0061] Crofts HS , Muggleton NG , Bowditch AP , Pearce PC , Nutt DJ , Scott EA. 1999 Home cage presentation of complex discrimination tasks to marmosets and rhesus monkeys. Lab Anim 33:207–214. 10780838 10.1258/002367799780578174

[dneu22446-bib-0062] Cubillo A , Halari R , Smith A , Taylor E , Rubia K. 2012 A review of fronto‐striatal and fronto‐cortical brain abnormalities in children and adults with Attention Deficit Hyperactivity Disorder (ADHD) and new evidence for dysfunction in adults with ADHD during motivation and attention. Cortex 48:194–215. 21575934 10.1016/j.cortex.2011.04.007

[dneu22446-bib-0563] Davidson MC , Amso D , Anderson LC , Diamond A . 2006 Development of cognitive control and executive functions from 4 to 13 years: Evidence from manipulations of memory, inhibition, and task switching. Neuropsychologia 44:2037–2078. 16580701 10.1016/j.neuropsychologia.2006.02.006 PMC1513793

[dneu22446-bib-0063] Davis C , Woodside DB. 2002 Sensitivity to the rewarding effects of food and exercise in the eating disorders. Compr Psychiatry 43:189–194. 11994836 10.1053/comp.2002.32356

[dneu22446-bib-0064] Davis M , Walker DL , Miles L , Grillon C. 2010 Phasic vs sustained fear in rats and humans: Role of the extended amygdala in fear vs anxiety. Neuropsychopharmacology 35:105–135. 19693004 10.1038/npp.2009.109 PMC2795099

[dneu22446-bib-0065] Del Campo N , Chamberlain SR , Sahakian BJ , Robbins TW. 2011 The roles of dopamine and noradrenaline in the pathophysiology and treatment of attention‐deficit/hyperactivity disorder. Biol Psychiatry 69:e145–e157. 21550021 10.1016/j.biopsych.2011.02.036

[dneu22446-bib-0066] Delahunty A , Morice R , Frost B. 1993 Specific cognitive flexibility rehabilitation in schizophrenia. Psychol Med 23:221–227. 8097331 10.1017/s0033291700039015

[dneu22446-bib-0067] Denk F , Walton ME , Jennings KA , Sharp T , Rushworth MFS , Bannerman DM. 2005 Differential involvement of serotonin and dopamine systems in cost‐benefit decisions about delay or effort. Psychopharmacology (Berl) 179:587–596. 15864561 10.1007/s00213-004-2059-4

[dneu22446-bib-0068] Dettling AC , Feldon J , Pryce CR. 2002 Repeated parental deprivation in the infant common marmoset (Callithrix jacchus, primates) and analysis of its effects on early development. Biol Psychiatry 52:1037–1046. 12460687 10.1016/s0006-3223(02)01460-9

[dneu22446-bib-0069] Dettling AC , Schnell CR , Maier C , Feldon J , Pryce CR. 2007 Behavioral and physiological effects of an infant‐neglect manipulation in a bi‐parental, twinning primate: Impact is dependent on familial factors. Psychoneuroendocrinology 32:331–349. 17363175 10.1016/j.psyneuen.2007.01.005

[dneu22446-bib-0070] Dias R , Robbins TW , Roberts AC. 1996a Primate analogue of the Wisconsin Card Sorting Test: Effects of excitotoxic lesions of the prefrontal cortex in the marmoset. Behav Neurosci 110:872–886. 8918991 10.1037//0735-7044.110.5.872

[dneu22446-bib-0071] Dias R , Robbins TW , Roberts AC. 1996b Dissociation in prefrontal cortex of affective and attentional shifts. Nature 380:69–72. 8598908 10.1038/380069a0

[dneu22446-bib-0072] Dichter GS , Smoski MJ , Kampov‐Polevoy AB , Gallop R , Garbutt JC. 2010 Unipolar depression does not moderate responses to the Sweet Taste Test. Depress Anxiety 27:859–863. 20336799 10.1002/da.20690 PMC2935496

[dneu22446-bib-0073] Dickson JM. 2006 Perceived consequences underlying approach goals and avoidance goals in relation to anxiety. Pers Individ Dif 41:1527–1538.

[dneu22446-bib-0074] Dowson JH , McLean A , Bazanis E , Toone B , Young S , Robbins TW , Sahakian BJ. 2004 Impaired spatial working memory in adults with attention‐deficit/hyperactivity disorder: Comparisons with performance in adults with borderline personality disorder and in control subjects. Acta Psychiatr Scand 110:45–54. 15180779 10.1111/j.1600-0447.2004.00292.x

[dneu22446-bib-0075] Drevets WC , Savitz J , Trimble M. 2008 The subgenual anterior cingulate cortex in mood disorders. CNS Spectr 13:663–681. 18704022 10.1017/s1092852900013754 PMC2729429

[dneu22446-bib-0076] Dudchenko PA , Talpos J , Young J , Baxter MG. 2013 Animal models of working memory: A review of tasks that might be used in screening drug treatments for the memory impairments found in schizophrenia. Neurosci Biobehav Rev 37:2111–2124. 22464948 10.1016/j.neubiorev.2012.03.003

[dneu22446-bib-0077] Duits P , Cath DC , Lissek S , Hox JJ , Hamm AO , Engelhard IM , van den Hout MA , Baas JMP. 2015 Updated meta‐analysis of classical fear conditioning in the anxiety disorders. Depress Anxiety 32:239–253. 25703487 10.1002/da.22353

[dneu22446-bib-0078] Dunlop BW , Nemeroff CB. 2007 The role of dopamine in the pathophysiology of depression. Arch Gen Psychiatry 64:327–337. 17339521 10.1001/archpsyc.64.3.327

[dneu22446-bib-0079] Durston S , Tottenham NT , Thomas KM , Davidson MC , Eigsti I‐M , Yang Y , Ulug AM , Casey B. 2003 Differential patterns of striatal activation in young children with and without ADHD. Biol Psychiatry 53:871–878. 12742674 10.1016/s0006-3223(02)01904-2

[dneu22446-bib-0080] Duvarci S , Bauer EP , Paré D. 2009 The bed nucleus of the stria terminalis mediates inter‐individual variations in anxiety and fear. J Neurosci 29:10357–10361. 19692610 10.1523/JNEUROSCI.2119-09.2009 PMC2741739

[dneu22446-bib-0081] Dymond S , Dunsmoor JE , Vervliet B , Roche B , Hermans D. 2015 Fear generalization in humans: Systematic review and implications for anxiety disorder research. Behav Ther 46:561–582. 26459838 10.1016/j.beth.2014.10.001

[dneu22446-bib-0082] Elliott R , McKenna PJ , Robbins TW , Sahakian BI. 1995 Specific neuropsychological deficits in schizophrenic patients with preserved intellectual function. Cogn Neuropsychiatry 25:619–630.

[dneu22446-bib-0083] Etkin A , Wager TD. 2007 Functional neuroimaging of anxiety: A meta‐analysis of emotional processing in PTSD, social anxiety disorder, and specific phobia. Am J Psychiatry 164:1476–1488. 17898336 10.1176/appi.ajp.2007.07030504 PMC3318959

[dneu22446-bib-0084] Etkin A , Egner T , Kalisch R. 2011 Emotional processing in anterior cingulate and medial prefrontal cortex. Trends Cogn Sci 15:85–93. 21167765 10.1016/j.tics.2010.11.004 PMC3035157

[dneu22446-bib-0085] Everett J , Lavoie K , Gagnon JF , Gosselin N. 2001 Performance of patients with schizophrenia on the Wisconsin Card Sorting Test (WCST). J Psychiatry Neurosci 26:123–130. 11291529 PMC1407748

[dneu22446-bib-0086] Fawcett J , Clark DC , Scheftner WA , Gibbons RD. 1983 Assessing anhedonia in psychiatric patients. Arch Gen Psychiatry 40:79–84. 6849623 10.1001/archpsyc.1983.01790010081010

[dneu22446-bib-0087] Feng C , Luo Y‐J , Krueger F. 2015 Neural signatures of fairness‐related normative decision making in the ultimatum game: A coordinate‐based meta‐analysis. Hum Brain Mapp 36:591–602. 25327760 10.1002/hbm.22649 PMC6869807

[dneu22446-bib-0088] Fontenelle L , Marques C , Engelhardt E , Versiani M. 2001 Impaired set‐shifting ability and therapeutic response in obsessive‐compulsive disorder. J Neuropsychiatry Clin Neurosci 13:508–510. 11748320 10.1176/jnp.13.4.508

[dneu22446-bib-0089] Fried R , Hirshfeld‐Becker D , Petty C , Batchelder H , Biederman J. 2015 How informative is the cantab to assess executive functioning in children with ADHD? A controlled study. J Atten Disord 19:468–475. 22923781 10.1177/1087054712457038

[dneu22446-bib-0090] Gard DE , Fisher M , Garrett C , Genevsky A , Vinogradov S. 2009 Motivation and its relationship to neurocognition, social cognition, and functional outcome in schizophrenia. Schizophr Res 115:74–81. 19783407 10.1016/j.schres.2009.08.015 PMC2757502

[dneu22446-bib-0091] Gard DE , Sanchez AH , Cooper K , Fisher M , Garrett C , Vinogradov S. 2014 Do people with schizophrenia have difficulty anticipating pleasure, engaging in effortful behavior, or both?. J Abnorm Psychol 123:771–782. 25133986 10.1037/abn0000005 PMC4227944

[dneu22446-bib-0092] Gillan CM , Papmeyer M , Morein‐Zamir S , Sahakian BJ , Fineberg NA , Robbins TW , de Wit S. 2011 Disruption in the balance between goal‐directed behavior and habit learning in obsessive‐compulsive disorder. Am J Psychiatry 168:718–726. 21572165 10.1176/appi.ajp.2011.10071062 PMC3533260

[dneu22446-bib-0094] Gorman JM , Korotzer A , Su G. 2014 Efficacy comparison of escitalopram and citalopram in the treatment of major depressive disorder: Pooled analysis of placebo‐controlled trials. CNS Spectr 7:40–44. 10.1017/s1092852900028595 15131492

[dneu22446-bib-0095] Gotlib IH , Krasnoperova E , Yue DN , Joormann J. 2004 Attentional biases for negative interpersonal stimuli in clinical depression. J Abnorm Psychol 113:121–135. 14992665 10.1037/0021-843X.113.1.121

[dneu22446-bib-0096] Graybiel AM , Rauch SL. 2000 Toward a neurobiology of obsessive‐compulsive disorder. Neuron 28:343–347. 11144344 10.1016/s0896-6273(00)00113-6

[dneu22446-bib-0097] Green MF. 1996 What are the functional consequences of neurocognitive deficits in schizophrenia?. Am J Psychiatry 153:321–330. 8610818 10.1176/ajp.153.3.321

[dneu22446-bib-0098] Grupe DW , Nitschke JB. 2013 Uncertainty and anticipation in anxiety: An integrated neurobiological and psychological perspective. Nat Rev Neurosci 14:488–501. 23783199 10.1038/nrn3524 PMC4276319

[dneu22446-bib-0099] Guyer AE , Lau JYF , McClure‐Tone EB , Parrish J , Shiffrin ND , Reynolds RC , Chen G , Blair RJR , Leibenluft E , Fox NA , et al. 2008 Amygdala and ventrolateral prefrontal cortex function during anticipated peer evaluation in pediatric social anxiety. Arch Gen Psychiatry 65:1303–1312. 18981342 10.1001/archpsyc.65.11.1303 PMC2717208

[dneu22446-bib-0100] Hampshire A , Owen AM. 2006 Fractionating attentional control using event‐related fMRI. Cereb Cortex 16:1679–1689. 16436686 10.1093/cercor/bhj116

[dneu22446-bib-0101] Harmer CJ , Cowen PJ. 2013 "It"s the way that you look at it’–a cognitive neuropsychological account of SSRI action in depression. Philos Trans R Soc Lond B Biol Sci 368:20120407. 23440467 10.1098/rstb.2012.0407 PMC3638386

[dneu22446-bib-0102] Harmer CJ , Mackay CE , Reid CB , Cowen PJ , Goodwin GM. 2006 Antidepressant drug treatment modifies the neural processing of nonconscious threat cues. Biol Psychiatry 59:816–820. 16460693 10.1016/j.biopsych.2005.10.015

[dneu22446-bib-0103] Heerey EA , Gold JM. 2007 Patients with schizophrenia demonstrate dissociation between affective experience and motivated behavior. J Abnorm Psychol 116:268–278. 17516760 10.1037/0021-843X.116.2.268

[dneu22446-bib-0104] Heller AS , Johnstone T , Shackman AJ , Light SN , Peterson MJ , Kolden GG , Kalin NH , Davidson RJ. 2009 Reduced capacity to sustain positive emotion in major depression reflects diminished maintenance of fronto‐striatal brain activation. Proc Natl Acad Sci U S A 106:22445–22450. 20080793 10.1073/pnas.0910651106 PMC2796908

[dneu22446-bib-0105] Herry C , Ciocchi S , Senn V , Demmou L , Müller C , Lüthi A. 2008 Switching on and off fear by distinct neuronal circuits. Nature 454:600–606. 18615015 10.1038/nature07166

[dneu22446-bib-0106] Hirsch C , Mathews A. 1997 Interpretative inferences when reading about emotional events. Behav Res Ther 35:1123–1132. 9465445

[dneu22446-bib-0107] Holland G. 1999 Amygdala circuitry in attentional and representational processes. Trends Cogn Sci 3:65–73. 10234229 10.1016/s1364-6613(98)01271-6

[dneu22446-bib-0108] Insel T , Cuthbert B , Garvey M , Heinssen R , Pine DS , Quinn K , Sanislow C , Wang P. 2010 Research domain criteria (RDoC): Toward a new classification framework for research on mental disorders. Am J Psychiatry 167:748–751. 20595427 10.1176/appi.ajp.2010.09091379

[dneu22446-bib-0109] Isella V , Iurlaro S , Piolti R , Ferrarese C , Frattola L , Appollonio I , Melzi P , Grimaldi M. 2003 Physical anhedonia in Parkinson's disease. J Neurol Neurosurg Psychiatry 74:1308–1311. 12933942 10.1136/jnnp.74.9.1308 PMC1738679

[dneu22446-bib-0110] Izquierdo A , Suda RK , Murray EA. 2005 Comparison of the effects of bilateral orbital prefrontal cortex lesions and amygdala lesions on emotional responses in rhesus monkeys. J Neurosci 25:8534–8542. 16162935 10.1523/JNEUROSCI.1232-05.2005 PMC6725674

[dneu22446-bib-0111] Izumi A , Tsuchida J , Yamaguchi C. 2012 Effects of rearing conditions on early visual development in common marmosets. Dev Psychobiol 54:700–705. 22127827 10.1002/dev.20619

[dneu22446-bib-0112] Jones PB. 2013 Adult mental health disorders and their age at onset. Br J Psychiatry Suppl 54:s5–s10. 10.1192/bjp.bp.112.119164 23288502

[dneu22446-bib-0113] Jones B , Mishkin M. 1972 Limbic lesions and the problem of stimulus—Reinforcement associations. Exp Neurol 36:362–377. 4626489 10.1016/0014-4886(72)90030-1

[dneu22446-bib-0114] Joyce E. 2002 Executive dysfunction in first‐episode schizophrenia and relationship to duration of untreated psychosis: The West London Study. Br J Psychiatry 181:38s–344. 10.1192/bjp.181.43.s38 12271799

[dneu22446-bib-0115] Joyce EM , Hutton SB , Mutsatsa SH , Barnes TRE. 2005 Cognitive heterogeneity in first‐episode schizophrenia. Br J Psychiatry 187:516–522. 16319403 10.1192/bjp.187.6.516

[dneu22446-bib-0116] Kalin N , Shelton S. 1989 Defensive behaviors in infant rhesus monkeys: Environmental cues and neurochemical regulation. Science 243:1718–1721. 2564702 10.1126/science.2564702

[dneu22446-bib-0117] Kalin NH , Shelton SE , Davidson RJ. 2007 Role of the primate orbitofrontal cortex in mediating anxious temperament. Biol Psychiatry 62:1134–1139. 17643397 10.1016/j.biopsych.2007.04.004 PMC4523234

[dneu22446-bib-0118] Kaplan G , Rogers LJ. 2006 Head‐cocking as a form of exploration in the common marmoset and its development. Dev Psychobiol 48:551–560. 17016839 10.1002/dev.20155

[dneu22446-bib-0819] Keefe RS , Silva SG , Perkins DO , Lieberman JA . 1999 The effects of atypical antipsychotic drugs on neurocognitive impairment in schizophrenia: a review and meta-analysis. Schizophr Bull 25:201–222. 10416727 10.1093/oxfordjournals.schbul.a033374

[dneu22446-bib-0119] Keedwell PA , Andrew C , Williams SCR , Brammer MJ , Phillips ML. 2005 The neural correlates of anhedonia in major depressive disorder. Biol Psychiatry 58:843–853. 16043128 10.1016/j.biopsych.2005.05.019

[dneu22446-bib-0120] Keers R , Uher R , Huezo‐Diaz P , Smith R , Jaffee S , Rietschel M , Henigsberg N , Kozel D , Mors O , Maier W , et al. 2011 Interaction between serotonin transporter gene variants and life events predicts response to antidepressants in the GENDEP project. Pharmacogenomics J 11:138–145. 20212518 10.1038/tpj.2010.14

[dneu22446-bib-0121] Kempton S , Vance A , Maruff P , Luk E , Costin J , Pantelis C. 1999 Executive function and attention deficit hyperactivity disorder: Stimulant medication and better executive function performance in children. Psychol Med 29:527–538. 10405075 10.1017/s0033291799008338

[dneu22446-bib-0122] Kessler RC , Berglund P , Demler O , Jin R , Merikangas KR , Walters EE. 2005 Lifetime prevalence and age‐of‐onset distributions of DSM‐IV disorders in the National Comorbidity Survey Replication. Arch Gen Psychiatry 62:593–602. 15939837 10.1001/archpsyc.62.6.593

[dneu22446-bib-0123] Kessler RC , Aguilar‐Gaxiola S , Alonso J , Chatterji S , Lee S , Ormel J , Üstün TB , Wang PS. 2009 The global burden of mental disorders: An update from the WHO World Mental Health (WMH) Surveys. Epidemiol Psichiatr Soc 18:23–33. 19378696 10.1017/s1121189x00001421 PMC3039289

[dneu22446-bib-0124] Khawaja IS , Westermeyer JJ , Gajwani P , Feinstein RE. 2009 Depression and coronary artery disease: The association, mechanisms, and therapeutic implications. Psychiatry (Edgmont) 6:38–51. PMC2719442 19724742

[dneu22446-bib-0125] Killgore WDS , Yurgelun‐Todd DA. 2005 Social anxiety predicts amygdala activation in adolescents viewing fearful faces. Neuroreport 16:1671–1675. 16189475 10.1097/01.wnr.0000180143.99267.bd

[dneu22446-bib-0126] Klingberg T , Forssberg H , Westerberg H. 2002 Training of working memory in children with ADHD. J Clin Exp Neuropsychol 24:781–791. 12424652 10.1076/jcen.24.6.781.8395

[dneu22446-bib-0127] Klingberg T , Fernell E , Olesen PJ , Johnson M , Gustafsson P , Dahlström K , Gillberg CG , Forssberg H , Westerberg H. 2005 Computerized training of working memory in children with ADHD–a randomized, controlled trial. J Am Acad Child Adolesc Psychiatry 44:177–186. 15689731 10.1097/00004583-200502000-00010

[dneu22446-bib-0128] Koenigs M , Grafman J. 2009 The functional neuroanatomy of depression: Distinct roles for ventromedial and dorsolateral prefrontal cortex. Behav Brain Res 201:239–243. 19428640 10.1016/j.bbr.2009.03.004 PMC2680780

[dneu22446-bib-0129] Lau JYF , Lissek S , Nelson EE , Lee Y , Roberson‐Nay R , Poeth K , Jenness J , Ernst M , Grillon C , Pine DS. 2008 Fear conditioning in adolescents with anxiety disorders: Results from a novel experimental paradigm. J Am Acad Child Adolesc Psychiatry 47:94–102. 18174830 10.1097/chi.0b01e31815a5f01 PMC2788509

[dneu22446-bib-0130] Lawrence NS , Wooderson S , Mataix‐Cols D , David R , Speckens A , Phillips ML. 2006 Decision making and set shifting impairments are associated with distinct symptom dimensions in obsessive‐compulsive disorder. Neuropsychology 20:409–419. 16846259 10.1037/0894-4105.20.4.409

[dneu22446-bib-0131] LeDoux JE. 2000 Emotion circuits in the brain. Annu Rev Neurosci 23:155–184. 10845062 10.1146/annurev.neuro.23.1.155

[dneu22446-bib-0132] Lee J , Park S. 2005 Working memory impairments in schizophrenia: A meta‐analysis. J Abnorm Psychol 114:599–611. 16351383 10.1037/0021-843X.114.4.599

[dneu22446-bib-0133] Leeson VC , Robbins TW , Matheson E , Hutton SB , Ron MA , Barnes TRE , Joyce EM. 2009 Discrimination learning, reversal, and set‐shifting in first‐episode schizophrenia: Stability over six years and specific associations with medication type and disorganization syndrome. Biol Psychiatry 66:586–593. 19576575 10.1016/j.biopsych.2009.05.016 PMC2734076

[dneu22446-bib-0134] Lenartowicz A , Delorme A , Walshaw PD , Cho AL , Bilder RM , McGough JJ , McCracken JT , Makeig S , Loo SK. 2014 Electroencephalography correlates of spatial working memory deficits in attention‐deficit/hyperactivity disorder: Vigilance, encoding, and maintenance. J Neurosci 34:1171–1182. 24453310 10.1523/JNEUROSCI.1765-13.2014 PMC3898282

[dneu22446-bib-0135] Lesch K‐P , Bengel D , Heils A , Sabol SZ , Greenberg BD , Petri S , Benjamin J , Muller CR , Hamer DH , Murphy DL. 1996 Association of anxiety‐related traits with a polymorphism in the serotonin transporter gene regulatory region. Science 274:1527–1531. 8929413 10.1126/science.274.5292.1527

[dneu22446-bib-0136] Lissek S. 2012 Toward an account of clinical anxiety predicated on basic, neurally mapped mechanisms of Pavlovian fear‐learning: The case for conditioned overgeneralization. Depress Anxiety 29:257–263. 22447565 10.1002/da.21922 PMC4194209

[dneu22446-bib-0137] Lissek S , Levenson J , Biggs AL , Johnson LL , Ameli R , Pine DS , Grillon C. 2008 Elevated fear conditioning to socially relevant unconditioned stimuli in social anxiety disorder. Am J Psychiatry 165:124–132. 18006874 10.1176/appi.ajp.2007.06091513 PMC2538574

[dneu22446-bib-0138] Lissek S , Kaczkurkin AN , Rabin S , Geraci M , Pine DS , Grillon C. 2014 Generalized anxiety disorder is associated with overgeneralization of classically conditioned fear. Biol Psychiatry 75:909–915. 24001473 10.1016/j.biopsych.2013.07.025 PMC3938992

[dneu22446-bib-0139] Luciana M. 2003 Practitioner Review: Computerized assessment of neuropsychological function in children: Clinical and research applications of the Cambridge Neuropsychological Testing Automated Battery (CANTAB). J Child Psychol Psychiatry 44:649–663. 12831110 10.1111/1469-7610.00152

[dneu22446-bib-0140] Lysaker P , Bell M , Beam‐Goulet J. 1995 Wisconsin card sorting test and work performance in schizophrenia. Psychiatry Res 56:45–51. 7792341 10.1016/0165-1781(94)02641-u

[dneu22446-bib-0141] MacDougall M , Nummela SU , Coop S , Disney A , Mitchell JF , Miller CT. 2016 Optogenetic manipulation of neural circuits in awake marmosets. J Neurophysiol, in press. 10.1152/jn.00197.2016 PMC5023415 27334951

[dneu22446-bib-0142] Machado CJ , Bachevalier J. 2008 Behavioral and hormonal reactivity to threat: Effects of selective amygdala, hippocampal or orbital frontal lesions in monkeys. Psychoneuroendocrinology 33:926–941. 18650022 10.1016/j.psyneuen.2008.04.012 PMC2564854

[dneu22446-bib-0143] Mackintosh NJ , Little L. 1969 Intradimensional and extradimensional shift learning by pigeons. Psychon Sci 14:5–6.

[dneu22446-bib-0144] Malmo HP. 1974 On frontal lobe functions: Psychiatric patient controls. Cortex 10:231–237. 16295096 10.1016/s0010-9452(74)80015-8

[dneu22446-bib-0145] Manes F , Sahakian B , Clark L , Rogers R , Antoun N , Aitken M , Robbins T. 2002 Decision‐making processes following damage to the prefrontal cortex. Brain 125:624–639. 11872618 10.1093/brain/awf049

[dneu22446-bib-0146] Manoach DS. 2003 Prefrontal cortex dysfunction during working memory performance in schizophrenia: Reconciling discrepant findings. Schizophr Res 60:285–298. 12591590 10.1016/s0920-9964(02)00294-3

[dneu22446-bib-0147] Mantella RC , Butters MA , Dew MA , Mulsant BH , Begley AE , Tracey B , Shear MK , Reynolds CF , Lenze EJ. 2007 Cognitive impairment in late‐life generalized anxiety disorder. Am J Geriatr Psychiatry 15:673–679. 17426260 10.1097/JGP.0b013e31803111f2

[dneu22446-bib-0148] Maren S. 2001 Neurobiology of Pavlovian fear conditioning. Annu Rev Neurosci 24:897–931. 11520922 10.1146/annurev.neuro.24.1.897

[dneu22446-bib-0149] Markou A , Koob GF. 1991 Postcocaine anhedonia. An animal model of cocaine withdrawal. Neuropsychopharmacology 4:17–26. 2003866

[dneu22446-bib-0150] Martinussen R , Hayden J , Hogg‐Johnson S , Tannock R. 2005 A meta‐analysis of working memory impairments in children with attention‐deficit/hyperactivity disorder. J Am Acad Child Adolesc Psychiatry 44:377–384. 15782085 10.1097/01.chi.0000153228.72591.73

[dneu22446-bib-0151] Marumo K , Takizawa R , Kinou M , Kawasaki S , Kawakubo Y , Fukuda M , Kasai K. 2014 Functional abnormalities in the left ventrolateral prefrontal cortex during a semantic fluency task, and their association with thought disorder in patients with schizophrenia. Neuroimage 85 Pt 1:518–526. 23624170 10.1016/j.neuroimage.2013.04.050

[dneu22446-bib-0152] Mashiko H , Yoshida AC , Kikuchi SS , Niimi K , Takahashi E , Aruga J , Okano H , Shimogori T. 2012 Comparative anatomy of marmoset and mouse cortex from genomic expression. J Neurosci 32:5039–5053. 22496550 10.1523/JNEUROSCI.4788-11.2012 PMC6622108

[dneu22446-bib-0153] Mattfeld AT , Whitfield‐Gabrieli S , Biederman J , Spencer T , Brown A , Fried R , Gabrieli JDE. 2015 Dissociation of working memory impairments and attention‐deficit/hyperactivity disorder in the brain. NeuroImage Clin 10:274–282. 26900567 10.1016/j.nicl.2015.12.003 PMC4723732

[dneu22446-bib-0154] McFarland BR , Klein DN. 2009 Emotional reactivity in depression: Diminished responsiveness to anticipated reward but not to anticipated punishment or to nonreward or avoidance. Depress Anxiety 26:117–122. 18972567 10.1002/da.20513

[dneu22446-bib-0155] McKirdy J , Sussmann JED , Hall J , Lawrie SM , Johnstone EC , McIntosh AM. 2009 Set shifting and reversal learning in patients with bipolar disorder or schizophrenia. Psychol Med 39:1289–1293. 19105856 10.1017/S0033291708004935

[dneu22446-bib-0156] Mehta MA , Goodyer IM , Sahakian BJ. 2004 Methylphenidate improves working memory and set‐shifting in AD/HD: Relationships to baseline memory capacity. J Child Psychol Psychiatry 45:293–305. 14982243 10.1111/j.1469-7610.2004.00221.x

[dneu22446-bib-0157] Menzies L , Chamberlain SR , Laird AR , Thelen SM , Sahakian BJ , Bullmore ET. 2008 Integrating evidence from neuroimaging and neuropsychological studies of obsessive‐compulsive disorder: The orbitofronto‐striatal model revisited. Neurosci Biobehav Rev 32:525–549. 18061263 10.1016/j.neubiorev.2007.09.005 PMC2889493

[dneu22446-bib-0158] Mikheenko Y , Shiba Y , Sawiak S , Braesicke K , Cockcroft G , Clarke H , Roberts AC. 2015 Serotonergic, brain volume and attentional correlates of trait anxiety in primates. Neuropsychopharmacology 40:1395–1404. 25586542 10.1038/npp.2014.324 PMC4397084

[dneu22446-bib-0159] Milad MR , Rauch SL. 2007 The role of the orbitofrontal cortex in anxiety disorders. Ann N Y Acad Sci 1121:546–561. 17698998 10.1196/annals.1401.006

[dneu22446-bib-0160] Milner B. 1963 Effects of different brain lesions on card sorting. Arch Neurol 9:90.

[dneu22446-bib-0961] Mishara AL , Goldberg TE . 2004 A meta-analysis and critical review of the effects of conventional neuroleptic treatment on cognition in schizophrenia: opening a closed book. Biol Psychiatry 55:1013–1022. 15121486 10.1016/j.biopsych.2004.01.027

[dneu22446-bib-0161] Miyamoto S , Duncan GE , Marx CE , Lieberman JA. 2005 Treatments for schizophrenia: A critical review of pharmacology and mechanisms of action of antipsychotic drugs. Mol Psychiatry 10:79–104. 15289815 10.1038/sj.mp.4001556

[dneu22446-bib-0162] Mogg K , Bradley BP. 2005 Attentional bias in generalized anxiety disorder versus depressive disorder. Cognit Ther Res 29:29–45.

[dneu22446-bib-0163] Mogg K , Bradley BP , Dixon C , Fisher S , Twelftree H , McWilliams A. 2000 Trait anxiety, defensiveness and selective processing of threat: An investigation using two measures of attentional bias. Pers Individ Dif 28:1063–1077.

[dneu22446-bib-0164] Monk CS , Nelson EE , McClure EB , Mogg K , Bradley BP , Leibenluft E , Blair RJR , Chen G , Charney DS , Ernst M , et al. 2006 Ventrolateral prefrontal cortex activation and attentional bias in response to angry faces in adolescents with generalized anxiety disorder. Am J Psychiatry 163:1091–1097. 16741211 10.1176/ajp.2006.163.6.1091

[dneu22446-bib-0165] Morgiève M , N'Diaye K , Haynes WIA , Granger B , Clair A‐H , Pelissolo A , Mallet L. 2014 Dynamics of psychotherapy‐related cerebral haemodynamic changes in obsessive compulsive disorder using a personalized exposure task in functional magnetic resonance imaging. Psychol Med 44:1461–1473. 24001313 10.1017/S0033291713002237

[dneu22446-bib-0166] Morice R. 1990 Cognitive inflexibility and pre‐frontal dysfunction in schizophrenia and mania. Br J Psychiatry 157:50–54. 2397362 10.1192/bjp.157.1.50

[dneu22446-bib-0167] Morris JS , Ohman A , Dolan RJ. 1999 A subcortical pathway to the right amygdala mediating "unseen" fear. Proc Natl Acad Sci 96:1680–1685. 9990084 10.1073/pnas.96.4.1680 PMC15559

[dneu22446-bib-0168] Mueller EM , Nguyen J , Ray WJ , Borkovec TD. 2010 Future‐oriented decision‐making in Generalized Anxiety Disorder is evident across different versions of the Iowa Gambling Task. J Behav Ther Exp Psychiatry 41:165–171. 20060098 10.1016/j.jbtep.2009.12.002

[dneu22446-bib-0169] Munafò MR , Freimer NB , Ng W , Ophoff R , Veijola J , Miettunen J , Järvelin M‐R , Taanila A , Flint J. 2009 5‐HTTLPR genotype and anxiety‐related personality traits: A meta‐analysis and new data. Am J Med Genet B Neuropsychiatr Genet 150B:271–281. 18546120 10.1002/ajmg.b.30808 PMC2819421

[dneu22446-bib-0170] Murphy FC , Sahakian BJ , Rubinsztein JS , Michael A , Rogers RD , Robbins TW , Paykel ES. 1999 Emotional bias and inhibitory control processes in mania and depression. Psychol Med 29:1307–1321. 10616937 10.1017/s0033291799001233

[dneu22446-bib-0171] Murphy FC , Nimmo‐Smith I , Lawrence AD. 2003 Functional neuroanatomy of emotions: A meta‐analysis. Cogn Affect Behav Neurosci 3:207–233. 14672157 10.3758/cabn.3.3.207

[dneu22446-bib-0172] Murphy SE , Norbury R , O'Sullivan U , Cowen PJ , Harmer CJ. 2009 Effect of a single dose of citalopram on amygdala response to emotional faces. Br J Psychiatry 194:535–540. 19478294 10.1192/bjp.bp.108.056093 PMC2802527

[dneu22446-bib-0173] Murphy SE , Norbury R , Godlewska BR , Cowen PJ , Mannie ZM , Harmer CJ , Munafò MR. 2013 The effect of the serotonin transporter polymorphism (5‐HTTLPR) on amygdala function: A meta‐analysis. Mol Psychiatry 18:512–520. 22488255 10.1038/mp.2012.19

[dneu22446-bib-0174] Murray EA. 2007 The amygdala, reward and emotion. Trends Cogn Sci 11:489–497. 17988930 10.1016/j.tics.2007.08.013

[dneu22446-bib-0175] Myers‐Schulz B , Koenigs M. 2012 Functional anatomy of ventromedial prefrontal cortex: Implications for mood and anxiety disorders. Mol Psychiatry 17:132–141. 21788943 10.1038/mp.2011.88 PMC3937071

[dneu22446-bib-0176] Nakao T , Nakagawa A , Yoshiura T , Nakatani E , Nabeyama M , Yoshizato C , Kudoh A , Tada K , Yoshioka K , Kawamoto M , et al. 2005 Brain activation of patients with obsessive‐compulsive disorder during neuropsychological and symptom provocation tasks before and after symptom improvement: A functional magnetic resonance imaging study. Biol Psychiatry 57:901–910. 15820711 10.1016/j.biopsych.2004.12.039

[dneu22446-bib-0177] Nakako T , Murai T , Ikejiri M , Ishiyama T , Taiji M , Ikeda K. 2013 Effects of a dopamine D1 agonist on ketamine‐induced spatial working memory dysfunction in common marmosets. Behav Brain Res 249:109–115. 23608483 10.1016/j.bbr.2013.04.012

[dneu22446-bib-0178] Nugent NR , Tyrka AR , Carpenter LL , Price LH. 2011 Gene–environment interactions: Early life stress and risk for depressive and anxiety disorders. Psychopharmacology (Berl) 214:175–196. 21225419 10.1007/s00213-010-2151-x PMC3615637

[dneu22446-bib-0179] Nutt D , Demyttenaere K , Janka Z , Aarre T , Bourin M , Canonico PL , Carrasco JL , Stahl S. 2007 The other face of depression, reduced positive affect: The role of catecholamines in causation and cure. J Psychopharmacol 21:461–471. 17050654 10.1177/0269881106069938

[dneu22446-bib-0180] Okasha A , Rafaat M , Mahallawy N , Nahas J , Seif El Dawla A , Sayed M , El Kholi S. 2000 Cognitive dysfunction in obsessive?compulsive disorder. Acta Psychiatr Scand 101:281–285. 10782547

[dneu22446-bib-0181] Ongur D , Price JL. 2000 The organization of networks within the orbital and medial prefrontal cortex of rats, monkeys and humans. Cereb Cortex 10:206–219. 10731217 10.1093/cercor/10.3.206

[dneu22446-bib-0182] Orban GA , Van Essen D , Vanduffel W. 2004 Comparative mapping of higher visual areas in monkeys and humans. Trends Cogn Sci 8:315–324. 15242691 10.1016/j.tics.2004.05.009

[dneu22446-bib-0183] Orfei MD , Piras F , Macci E , Caltagirone C , Spalletta G. 2013 The neuroanatomical correlates of cognitive insight in schizophrenia. Soc Cogn Affect Neurosci 8:418–423. 22287264 10.1093/scan/nss016 PMC3624955

[dneu22446-bib-0184] Owen AM , Downes JJ , Sahakian BJ , Polkey CE , Robbins TW. 1990 Planning and spatial working memory following frontal lobe lesions in man. Neuropsychologia 28:1021–1034. 2267054 10.1016/0028-3932(90)90137-d

[dneu22446-bib-0185] Pacella ML , Hruska B , Delahanty DL. 2013 The physical health consequences of PTSD and PTSD symptoms: A meta‐analytic review. J Anxiety Disord 27:33–46. 23247200 10.1016/j.janxdis.2012.08.004

[dneu22446-bib-0186] Pantelis C , Barber FZ , Barnes TR , Nelson HE , Owen AM , Robbins TW. 1999 Comparison of set‐shifting ability in patients with chronic schizophrenia and frontal lobe damage. Schizophr Res 37:251–270. 10403197 10.1016/s0920-9964(98)00156-x

[dneu22446-bib-0187] Peckham AD , McHugh RK , Otto MW. 2010 A meta‐analysis of the magnitude of biased attention in depression. Depress Anxiety 27:1135–1142. 21049527 10.1002/da.20755

[dneu22446-bib-0188] Pelizza L , Ferrari A. 2009 Anhedonia in schizophrenia and major depression: State or trait?. Ann Gen Psychiatry 8:22. 19811665 10.1186/1744-859X-8-22 PMC2764701

[dneu22446-bib-0189] Perna G , Favaron E , Di Bella D , Bussi R , Bellodi L. 2005 Antipanic efficacy of paroxetine and polymorphism within the promoter of the serotonin transporter gene. Neuropsychopharmacology 30:2230–2235. 16034444 10.1038/sj.npp.1300822

[dneu22446-bib-0190] Petrides M. 1995a Impairments on nonspatial self‐ordered and externally ordered working memory tasks after lesions of the mid‐dorsal part of the lateral frontal cortex in the monkey. J Neurosci 15:359–375. 7823141 10.1523/JNEUROSCI.15-01-00359.1995 PMC6578311

[dneu22446-bib-0191] Petrides M. 1995b Functional organization of the human frontal cortex for mnemonic processing. Evidence from neuroimaging studies. Ann N Y Acad Sci 769:85–96. 8595046 10.1111/j.1749-6632.1995.tb38133.x

[dneu22446-bib-0192] Petrides M. 2005 Lateral prefrontal cortex: Architectonic and functional organization. Philos Trans R Soc Lond B Biol Sci 360:781–795. 15937012 10.1098/rstb.2005.1631 PMC1569489

[dneu22446-bib-0193] Petrides M , Pandya DN. 2002 Comparative cytoarchitectonic analysis of the human and the macaque ventrolateral prefrontal cortex and corticocortical connection patterns in the monkey. Eur J Neurosci 16:291–310. 12169111 10.1046/j.1460-9568.2001.02090.x

[dneu22446-bib-0194] Petrides M , Pandya DN. 2007 Efferent association pathways from the rostral prefrontal cortex in the macaque monkey. J Neurosci 27:11573–11586. 17959800 10.1523/JNEUROSCI.2419-07.2007 PMC6673207

[dneu22446-bib-0195] Petrides M , Tomaiuolo F , Yeterian EH , Pandya DN. 2012 The prefrontal cortex: Comparative architectonic organization in the human and the macaque monkey brains. Cortex 48:46–57. 21872854 10.1016/j.cortex.2011.07.002

[dneu22446-bib-0196] Piper MC , Pinnell LE , Darrah J , Maguire T , Byrne PJ. 1992 Construction and validation of the Alberta Infant Motor Scale (AIMS). Can J Public Health 83 Suppl 2:S46–S50. 1468050

[dneu22446-bib-0197] Pirkola T , Tuulio‐Henriksson A , Glahn D , Kieseppä T , Haukka J , Kaprio J , Lönnqvist J , Cannon TD. 2005 Spatial working memory function in twins with schizophrenia and bipolar disorder. Biol Psychiatry 58:930–936. 16112657 10.1016/j.biopsych.2005.05.041

[dneu22446-bib-0198] Pistorio AL , Vintch B , Wang X. 2006 Acoustic analysis of vocal development in a New World primate, the common marmoset (Callithrix jacchus). J Acoust Soc Am 120:1655–1670. 17004487 10.1121/1.2225899

[dneu22446-bib-0199] Pontecorvo MJ , Sahgal A , Steckler T. 1996 Further developments in the measurement of working memory in rodents. Cogn Brain Res 3:205–213. 10.1016/0926-6410(96)00007-9 8806023

[dneu22446-bib-0200] Porcelli S , Fabbri C , Serretti A. 2012 Meta‐analysis of serotonin transporter gene promoter polymorphism (5‐HTTLPR) association with antidepressant efficacy. Eur Neuropsychopharmacol 22:239–258. 22137564 10.1016/j.euroneuro.2011.10.003

[dneu22446-bib-0201] Price JL , Drevets WC. 2010 Neurocircuitry of mood disorders. Neuropsychopharmacology 35:192–216. 19693001 10.1038/npp.2009.104 PMC3055427

[dneu22446-bib-0202] Pryce CR , Dettling AC , Spengler M , Schnell CR , Feldon J. 2004 Deprivation of parenting disrupts development of homeostatic and reward systems in marmoset monkey offspring. Biol Psychiatry 56:72–79. 15231438 10.1016/j.biopsych.2004.05.002

[dneu22446-bib-0203] Pu S , Nakagome K , Yamada T , Itakura M , Satake T , Ishida H , Nagata I , Kaneko K. 2013 Association between cognitive insight and prefrontal function during a cognitive task in schizophrenia: A multichannel near‐infrared spectroscopy study. Schizophr Res 150:81–87. 23953828 10.1016/j.schres.2013.07.048

[dneu22446-bib-0204] Purcell R , Maruff P , Kyrios M , Pantelis C. 1998 Cognitive deficits in obsessive–compulsive disorder on tests of frontal–striatal function. Biol Psychiatry 43:348–357. 9513750 10.1016/s0006-3223(97)00201-1

[dneu22446-bib-0205] Rauschecker JP , Scott SK. 2009 Maps and streams in the auditory cortex: Nonhuman primates illuminate human speech processing. Nat Neurosci 12:718–724. 19471271 10.1038/nn.2331 PMC2846110

[dneu22446-bib-0206] Reekie YL , Braesicke K , Man MS , Roberts AC. 2008 Uncoupling of behavioral and autonomic responses after lesions of the primate orbitofrontal cortex. Proc Natl Acad Sci U S A 105:9787–9792. 18621690 10.1073/pnas.0800417105 PMC2447863

[dneu22446-bib-0207] Reinhold JA , Mandos LA , Rickels K , Lohoff FW. 2011 Pharmacological treatment of generalized anxiety disorder. Expert Opin Pharmacother 12:2457–2467. 21950420 10.1517/14656566.2011.618496

[dneu22446-bib-0208] Remijnse PL , van den Heuvel OA , Nielen MMA , Vriend C , Hendriks G‐J , Hoogendijk WJG , Uylings HBM , Veltman DJ. 2013 Cognitive inflexibility in obsessive‐compulsive disorder and major depression is associated with distinct neural correlates. PLoS One 8:e59600. 23637737 10.1371/journal.pone.0059600 PMC3634812

[dneu22446-bib-0209] Risch N , Herrell R , Lehner T , Liang K‐Y , Eaves L , Hoh J , Griem A , Kovacs M , Ott J , Merikangas KR. 2009 Interaction between the serotonin transporter gene (5‐HTTLPR), stressful life events, and risk of depression: A meta‐analysis. JAMA 301:2462–2471. 19531786 10.1001/jama.2009.878 PMC2938776

[dneu22446-bib-0210] Robbins TW , Everitt BJ. 1992 Functions of dopamine in the dorsal and ventral striatum. Semin Neurosci 4:119–127.

[dneu22446-bib-0211] Roberts AC , Robbins TW , Everitt BJ. 1989 The effects of intradimensional and extradimensional shifts on visual discrimination learning in humans and non‐human primates. Q J Exp Psychol 40:321–341. 3145534

[dneu22446-bib-0212] Roberts AC , De Salvia MA , Wilkinson LS , Collins P , Muir JL , Everitt BJ , Robbins TW. 1994 6‐Hydroxydopamine lesions of the prefrontal cortex in monkeys enhance performance on an analog of the Wisconsin Card Sort Test: Possible interactions with subcortical dopamine. J Neurosci 14:2531–2544. 8182426 10.1523/JNEUROSCI.14-05-02531.1994 PMC6577476

[dneu22446-bib-0213] Robinson OJ , Cools R , Carlisi CO , Sahakian BJ , Drevets WC. 2012 Ventral striatum response during reward and punishment reversal learning in unmedicated major depressive disorder. Am J Psychiatry 169:152–159. 22420038 10.1176/appi.ajp.2011.11010137 PMC5648982

[dneu22446-bib-0214] Rock PL , Roiser JP , Riedel WJ , Blackwell AD. 2014 Cognitive impairment in depression: A systematic review and meta‐analysis. Psychol Med 44:2029–2040. 24168753 10.1017/S0033291713002535

[dneu22446-bib-0215] Rolland B , Amad A , Poulet E , Bordet R , Vignaud A , Bation R , Delmaire C , Thomas P , Cottencin O , Jardri R. 2015 Resting‐state functional connectivity of the nucleus accumbens in auditory and visual hallucinations in schizophrenia. Schizophr Bull 41:291–299. 25053649 10.1093/schbul/sbu097 PMC4266295

[dneu22446-bib-0216] Rotge J‐Y , Guehl D , Dilharreguy B , Cuny E , Tignol J , Bioulac B , Allard M , Burbaud P , Aouizerate B. 2008 Provocation of obsessive‐compulsive symptoms: A quantitative voxel‐based meta‐analysis of functional neuroimaging studies. J Psychiatry Neurosci 33:405–412. 18787662 PMC2527721

[dneu22446-bib-0217] Rudebeck PH , Murray EA. 2014 The orbitofrontal oracle: Cortical mechanisms for the prediction and evaluation of specific behavioral outcomes. Neuron 84:1143–1156. 25521376 10.1016/j.neuron.2014.10.049 PMC4271193

[dneu22446-bib-0218] Rudebeck PH , Walton ME , Smyth AN , Bannerman DM , Rushworth MFS. 2006 Separate neural pathways process different decision costs. Nat Neurosci 9:1161–1168. 16921368 10.1038/nn1756

[dneu22446-bib-0219] Rudebeck PH , Putnam PT , Daniels TE , Yang T , Mitz AR , Rhodes SEV , Murray EA. 2014 A role for primate subgenual cingulate cortex in sustaining autonomic arousal. Proc Natl Acad Sci 111:5391–5396. 24706828 10.1073/pnas.1317695111 PMC3986148

[dneu22446-bib-0220] Rygula R , Clarke HF , Cardinal RN , Cockcroft GJ , Xia J , Dalley JW , Robbins TW , Roberts AC. 2015 Role of central serotonin in anticipation of rewarding and punishing outcomes: Effects of selective amygdala or orbitofrontal 5‐ht depletion. Cereb Cortex 25:3064–3076. 24879752 10.1093/cercor/bhu102 PMC4537445

[dneu22446-bib-0221] Salamone J. 1997 Behavioral functions of nucleus accumbens dopamine: Empirical and conceptual problems with the anhedonia hypothesis. Neurosci Biobehav Rev 21:341–359. 9168269 10.1016/s0149-7634(96)00017-6

[dneu22446-bib-0222] Santangelo AM , Ito M , Shiba Y , Clarke HF , Schut EH , Cockcroft G , Ferguson‐Smith AC , Roberts AC. 2016 Novel primate model of serotonin transporter genetic polymorphisms associated with gene expression, anxiety and sensitivity to antidepressants. Neuropsychopharmacology 41:2366–2376. 26997299 10.1038/npp.2016.41 PMC4946067

[dneu22446-bib-0223] Sarsour K , Sheridan M , Jutte D , Nuru‐Jeter A , Hinshaw S , Boyce WT. 2011 Family socioeconomic status and child executive functions: The roles of language, home environment, and single parenthood. J Int Neuropsychol Soc 17:120–132. 21073770 10.1017/S1355617710001335

[dneu22446-bib-0224] Saxena S , Gorbis E , O'Neill J , Baker SK , Mandelkern MA , Maidment KM , Chang S , Salamon N , Brody AL , Schwartz JM , et al. 2009 Rapid effects of brief intensive cognitive‐behavioral therapy on brain glucose metabolism in obsessive‐compulsive disorder. Mol Psychiatry 14:197–205. 18180761 10.1038/sj.mp.4002134 PMC2893580

[dneu22446-bib-0225] Schlagenhauf F , Huys QJM , Deserno L , Rapp MA , Beck A , Heinze H‐J , Dolan R , Heinz A. 2014 Striatal dysfunction during reversal learning in unmedicated schizophrenia patients. Neuroimage 89:171–180. 24291614 10.1016/j.neuroimage.2013.11.034 PMC3991847

[dneu22446-bib-0226] Schoenbaum G , Roesch M. 2005 Orbitofrontal cortex, associative learning, and expectancies. Neuron 47:633–636. 16129393 10.1016/j.neuron.2005.07.018 PMC2628809

[dneu22446-bib-0227] Schoenbaum G , Saddoris MP , Stalnaker TA. 2007 Reconciling the roles of orbitofrontal cortex in reversal learning and the encoding of outcome expectancies. Ann N Y Acad Sci 1121:320–335. 17698988 10.1196/annals.1401.001 PMC2430624

[dneu22446-bib-0228] Schultz W. 2004 Neural coding of basic reward terms of animal learning theory, game theory, microeconomics and behavioural ecology. Curr Opin Neurobiol 14:139–147. 15082317 10.1016/j.conb.2004.03.017

[dneu22446-bib-0229] Schultz W , Tremblay L , Hollerman JR. 2000 Reward processing in primate orbitofrontal cortex and basal ganglia. Cereb Cortex 10:272–283. 10731222 10.1093/cercor/10.3.272

[dneu22446-bib-0230] Schultz W , Abe H , Lee D , Adamantidis A , Tsai H , Boutrel B , Zhang F , Stuber G , Budygin E , Touriño C , et al. 2015 Neuronal reward and decision signals: From theories to data. Physiol Rev 95:853–951. 26109341 10.1152/physrev.00023.2014 PMC4491543

[dneu22446-bib-0231] Schultz‐Darken N , Braun KM , Emborg ME. 2016 Neurobehavioral development of common marmoset monkeys. Dev Psychobiol 58:141–158. 26502294 10.1002/dev.21360 PMC4829073

[dneu22446-bib-0232] Sepehry AA , Potvin S , Élie R , Stip E. 2007 Selective serotonin reuptake inhibitor (ssri) add‐on therapy for the negative symptoms of schizophrenia. J Clin Psychiatry 68:604–610. 17474817 10.4088/jcp.v68n0417

[dneu22446-bib-0233] Sexton KA , Norton PJ , Walker JR , Norton GR. 2010 Hierarchical model of generalized and specific vulnerabilities in anxiety. Cogn Behav Ther 32:82–94. 10.1080/16506070302321 16291539

[dneu22446-bib-0234] Shelton RC , Tomarken AJ. 2001 Can recovery from depression be achieved?. Psychiatr Serv 52:1469–1478. 11684742 10.1176/appi.ps.52.11.1469

[dneu22446-bib-0235] Shiba Y , Kim C , Santangelo AM , Roberts AC. 2014a Lesions of either anterior orbitofrontal cortex or ventrolateral prefrontal cortex in marmoset monkeys heighten innate fear and attenuate active coping behaviors to predator threat. Front Syst Neurosci 8:250. 25653599 10.3389/fnsys.2014.00250 PMC4300917

[dneu22446-bib-0236] Shiba Y , Santangelo AM , Braesicke K , Agustín‐Pavón C , Cockcroft G , Haggard M , Roberts AC. 2014b Individual differences in behavioral and cardiovascular reactivity to emotive stimuli and their relationship to cognitive flexibility in a primate model of trait anxiety. Front Behav Neurosci 8:137. 24795587 10.3389/fnbeh.2014.00137 PMC4006051

[dneu22446-bib-0237] Shiba Y , Santangelo AM , Roberts AC. 2016 Beyond the medial regions of prefrontal cortex in the regulation of fear and anxiety. Front Syst Neurosci 10:12. 26941618 10.3389/fnsys.2016.00012 PMC4761915

[dneu22446-bib-0238] Shin LM , Rauch SL , Pitman RK. 2006 Amygdala, medial prefrontal cortex, and hippocampal function in PTSD. Ann N Y Acad Sci 1071:67–79. 16891563 10.1196/annals.1364.007

[dneu22446-bib-0239] Simpson HB , Rosen W , Huppert JD , Lin S‐H , Foa EB , Liebowitz MR. 2006 Are there reliable neuropsychological deficits in obsessive‐compulsive disorder?. J Psychiatr Res 40:247–257. 15950242 10.1016/j.jpsychires.2005.04.004

[dneu22446-bib-0240] Slattery DA , Markou A , Cryan JF. 2007 Evaluation of reward processes in an animal model of depression. Psychopharmacology (Berl) 190:555–568. 17177055 10.1007/s00213-006-0630-x

[dneu22446-bib-0241] Smoski MJ , Lynch TR , Rosenthal MZ , Cheavens JS , Chapman AL , Krishnan RR. 2008 Decision‐making and risk aversion among depressive adults. J Behav Ther Exp Psychiatry 39:567–576. 18342834 10.1016/j.jbtep.2008.01.004 PMC2590786

[dneu22446-bib-0242] Snaith RP , Hamilton M , Morley S , Humayan A , Hargreaves D , Trigwell P. 1995 A scale for the assessment of hedonic tone the Snaith‐Hamilton Pleasure Scale. Br J Psychiatry 167:99–103. 7551619 10.1192/bjp.167.1.99

[dneu22446-bib-0243] Stapelberg NJ , Hamilton‐Craig I , Neumann DL , Shum DH , McConnell H. 2012 Mind and heart: Heart rate variability in major depressive disorder and coronary heart disease ‐ a review and recommendations. Aust N Z J Psychiatry 46:946–957. 22528974 10.1177/0004867412444624

[dneu22446-bib-0244] Staring ABP , Van der Gaag M , Van den Berge M , Duivenvoorden HJ , Mulder CL. 2009 Stigma moderates the associations of insight with depressed mood, low self‐esteem, and low quality of life in patients with schizophrenia spectrum disorders. Schizophr Res 115:363–369. 19616414 10.1016/j.schres.2009.06.015

[dneu22446-bib-0245] Starkstein SE , Mizrahi R , Garau L. 2005 Specificity of symptoms of depression in alzheimer disease: A longitudinal analysis. Am J Geriatr Psychiatry 13:802–807. 16166410 10.1176/appi.ajgp.13.9.802

[dneu22446-bib-0246] Strakowski SM , Delbello MP , Adler CM. 2005 The functional neuroanatomy of bipolar disorder: A review of neuroimaging findings. Mol Psychiatry 10:105–116. 15340357 10.1038/sj.mp.4001585

[dneu22446-bib-0247] Strawn JR , Bitter SM , Weber WA , Chu W‐J , Whitsel RM , Adler C , Cerullo MA , Eliassen J , Strakowski SM , DelBello MP. 2012 Neurocircuitry of generalized anxiety disorder in adolescents: A pilot functional neuroimaging and functional connectivity study. Depress Anxiety 29:939–947. 22628125 10.1002/da.21961

[dneu22446-bib-0248] Stuhrmann A , Dohm K , Kugel H , Zwanzger P , Redlich R , Grotegerd D , Rauch AV , Arolt V , Heindel W , Suslow T , et al. 2013 Mood‐congruent amygdala responses to subliminally presented facial expressions in major depression: Associations with anhedonia. J Psychiatry Neurosci 38:249–258. 23171695 10.1503/jpn.120060 PMC3692722

[dneu22446-bib-0249] Tabibnia G , Satpute AB , Lieberman MD. 2008 The sunny side of fairness: Preference for fairness activates reward circuitry (and disregarding unfairness activates self‐control circuitry). Psychol Sci 19:339–347. 18399886 10.1111/j.1467-9280.2008.02091.x

[dneu22446-bib-0250] Takemoto A , Izumi A , Miwa M , Nakamura K. 2011 Development of a compact and general‐purpose experimental apparatus with a touch‐sensitive screen for use in evaluating cognitive functions in common marmosets. J Neurosci Methods 199:82–86. 21549751 10.1016/j.jneumeth.2011.04.029

[dneu22446-bib-0251] Taylor Tavares JV , Clark L , Furey ML , Williams GB , Sahakian BJ , Drevets WC. 2008 Neural basis of abnormal response to negative feedback in unmedicated mood disorders. Neuroimage 42:1118–1126. 18586109 10.1016/j.neuroimage.2008.05.049 PMC2745889

[dneu22446-bib-0252] Thoma P , Wiebel B , Daum I. 2007 Response inhibition and cognitive flexibility in schizophrenia with and without comorbid substance use disorder. Schizophr Res 92:168–180. 17399952 10.1016/j.schres.2007.02.004

[dneu22446-bib-0253] Tovote P , Fadok JP , Lüthi A. 2015 Neuronal circuits for fear and anxiety. Nat Rev Neurosci 16:317–331. 25991441 10.1038/nrn3945

[dneu22446-bib-0254] Treadway MT , Zald DH. 2011 Reconsidering anhedonia in depression: Lessons from translational neuroscience. Neurosci Biobehav Rev 35:537–555. 20603146 10.1016/j.neubiorev.2010.06.006 PMC3005986

[dneu22446-bib-0255] Trémeau F , Antonius D , Cacioppo JT , Ziwich R , Butler P , Malaspina D , Javitt DC. 2010 Anticipated, on‐line and remembered positive experience in schizophrenia. Schizophr Res 122:199–205. 19906511 10.1016/j.schres.2009.10.019

[dneu22446-bib-0256] Tyrer P , Baldwin D. 2006 Generalised anxiety disorder. Lancet (London, England) 368:2156–2166. 10.1016/S0140-6736(06)69865-6 17174708

[dneu22446-bib-0257] Vaghi MM , Vértes PE , Kitzbichler MG , Apergis‐Schoute AM , van der Flier FE , Fineberg NA , Sule A , Zaman R , Voon V , Kundu P , et al. 2016 Specific fronto‐striatal circuits for impaired cognitive flexibility and goal‐directed planning in Obsessive‐Compulsive Disorder: Evidence from resting‐state functional connectivity. Biol Psychiatry 0:1163–1171. 10.1016/j.biopsych.2016.08.009 PMC6020061 27769568

[dneu22446-bib-0258] Valerius G , Lumpp A , Kuelz A‐K , Freyer T , Voderholzer U. 2008 Reversal learning as a neuropsychological indicator for the neuropathology of obsessive compulsive disorder? A behavioral study. J Neuropsychiatry Clin Neurosci 20:210–218. 18451192 10.1176/jnp.2008.20.2.210

[dneu22446-bib-0259] Veale DM , Sahakian BJ , Owen AM , Marks IM. 2009 Specific cognitive deficits in tests sensitive to frontal lobe dysfunction in obsessive–compulsive disorder. Psychol Med 26:1261. 10.1017/s0033291700035984 8931172

[dneu22446-bib-0260] Vink M , Ramsey NF , Raemaekers M , Kahn RS. 2006 Striatal dysfunction in schizophrenia and unaffected relatives. Biol Psychiatry 60:32–39. 16603134 10.1016/j.biopsych.2005.11.026

[dneu22446-bib-0261] Walker SC , Robbins TW , Roberts AC. 2009 Response disengagement on a spatial self‐ordered sequencing task: Effects of regionally selective excitotoxic lesions and serotonin depletion within the prefrontal cortex. J Neurosci 29:6033–6041. 19420270 10.1523/JNEUROSCI.0312-09.2009 PMC4263250

[dneu22446-bib-0262] Walton ME , Behrens TEJ , Noonan MP , Rushworth MFS. 2011 Giving credit where credit is due: Orbitofrontal cortex and valuation in an uncertain world. Ann N Y Acad Sci 1239:14–24. 22145871 10.1111/j.1749-6632.2011.06257.x

[dneu22446-bib-0263] Waltz JA , Gold JM. 2007 Probabilistic reversal learning impairments in schizophrenia: Further evidence of orbitofrontal dysfunction. Schizophr Res 93:296–303. 17482797 10.1016/j.schres.2007.03.010 PMC2063592

[dneu22446-bib-0264] Wassum KM , Izquierdo A. 2015 The basolateral amygdala in reward learning and addiction. Neurosci Biobehav Rev 57:271–283. 26341938 10.1016/j.neubiorev.2015.08.017 PMC4681295

[dneu22446-bib-0265] Watkins LH , Sahakian BJ , Robertson MM , Veale DM , Rogers RD , Pickard KM , Aitken MRF , Robbins TW. 2005 Executive function in Tourette's syndrome and obsessive–compulsive disorder. Psychol Med 35:571–582. 15856727 10.1017/s0033291704003691

[dneu22446-bib-0266] Weinberger DR , Berman KF. 1996 Prefrontal function in schizophrenia: Confounds and controversies. Philos Trans R Soc Lond B Biol Sci 351:1495–1503. 8941961 10.1098/rstb.1996.0135

[dneu22446-bib-0267] Wessa M , Flor H. 2007 Failure of extinction of fear responses in posttraumatic stress disorder: Evidence from second‐order conditioning. Am J Psychiatry 164:1684–1692. 17974933 10.1176/appi.ajp.2007.07030525

[dneu22446-bib-0268] Westerberg H , Hirvikoski T , Forssberg H , Klingberg T. 2004 Visuo‐spatial working memory span: A sensitive measure of cognitive deficits in children with ADHD. Child Neuropsychol 10:155–161. 15590494 10.1080/09297040409609806

[dneu22446-bib-0269] Williams LM , Kemp AH , Felmingham K , Liddell BJ , Palmer DM , Bryant RA. 2007 Neural biases to covert and overt signals of fear: Dissociation by trait anxiety and depression. J Cogn Neurosci 19:1595–1608. 17854280 10.1162/jocn.2007.19.10.1595

[dneu22446-bib-0270] Wood SJ , Pantelis C , Proffitt T , Phillips LJ , Stuart GW , Buchanan JA , Mahony K , Brewer W , Smith DJ , McGorry PD. 2003 Spatial working memory ability is a marker of risk‐for‐psychosis. Psychol Med 33:1239–1247. 14580078 10.1017/s0033291703008067

[dneu22446-bib-0271] Yeterian EH , Pandya DN , Tomaiuolo F , Petrides M. 2012 The cortical connectivity of the prefrontal cortex in the monkey brain. Cortex 48:58–81. 21481342 10.1016/j.cortex.2011.03.004 PMC3161133

[dneu22446-bib-0272] Yokoyama C , Kawasaki A , Hayashi T , Onoe H. 2013 Linkage between the midline cortical serotonergic system and social behavior traits: Positron emission tomography studies of common marmosets. Cereb Cortex 23:2136–2145. 22791806 10.1093/cercor/bhs196

